# Laser‐Induced Chemical Patterning of Graphene‐Black Phosphorus Hybrids

**DOI:** 10.1002/chem.71127

**Published:** 2026-05-14

**Authors:** Jasmin Eisenkolb, Mhamed Assebban, Tobias Dierke, Janina Maultzsch, Andreas Hirsch, Frank Hauke

**Affiliations:** ^1^ Department of Chemistry and Pharmacy & Center of Advanced Materials and Processes (ZMP) Friedrich‐Alexander Universität Erlangen‐Nürnberg Fürth Germany; ^2^ Chair of Experimental Physics Friedrich‐Alexander‐Universität Erlangen‐Nürnberg Erlangen Germany

**Keywords:** black phosphorus, covalent functionalization, graphene, heterostructures, Raman spectroscopy

## Abstract

Black phosphorus (BP) and graphene are representatives of the class of two‐dimensional (2D) materials, each material with its own outstanding and versatile properties. Creating hybrid systems between those materials allows us to combine the best of both worlds, and by tailored chemical modification, these heterostructure assemblies can be engineered and fine‐tuned toward desired applications. This study systematically investigates laser‐induced covalent functionalization of BP nanosheets and graphene‐BP (G‐BP) van der Waals (vdW) heterostructures using dibenzoyl peroxide‐based precursors. Although pristine BP exhibits extreme sensitivity to laser irradiation, the formation of the G‐BP heterostructure successfully enabled detailed Raman spectroscopy investigations and the successful spatially‐resolved covalent functionalization of the graphene layer under mild laser conditions. Moreover, the G‐BP hybrids revealed thickness‐dependent intensity modulation of the graphene Raman bands due to interference effects, which was successfully modelled and exploited for the heterostructure characterization. The reversibility of the functional group attachment was investigated via temperature‐dependent (T‐dependent) Raman studies.

## Graphical abstract

Van der Waals (vdW)‐based heterostructures consisting of graphene and black phosphorus are fabricated using a dry transfer technique. These are subjected to laser light in the so‐called laser writing procedure to introduce functional, organic moieties to the superjacent graphene lattice, enabling the fabrication of sophisticated and highly functional hybrid architectures.

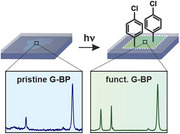

## 1 Introduction

Since the groundbreaking discovery and fabrication of graphene in 2004 by Novoselov and Geim [1], remarkable efforts have been dedicated to the exploration and development of novel two‐dimensional (2D) materials as they hold immense potential to revolutionize next‐generation technologies [2, 3]. Within this period, black phosphorus (BP) experienced its renaissance, being rediscovered as a 2D material with exceptional properties [4]. BP´s peculiar combination of a high charge carrier mobility, an excellent current on/off ratio and an unique in‐plane anisotropy, in combination with its tunable bandgap [5] renders BP an exquisite candidate for a broad range of applications in field‐effect transistors [6, 7], solar cells and various photovoltaic devices [8, 9, 10, 11], thermoelectronics [12, 13], sensors [14, 15], photodetectors [16, 17], saturable absorbers [18], and photocatalysis [19, 20, 21].

However, the intrinsic instability of BP under ambient conditions has significantly hindered implementations in large‐scale applications. Maintaining an inert atmosphere is associated with considerable efforts, and the subsequent operation of devices under such strict conditions is often impractical [22, 23, 24]. Thus far, to address this, various strategies have been explored to prevent reactive oxygen species from reaching the top‐most layer of BP. Among these, covering the nanosheets with inorganic coating layers is a facile and straightforward method, impeding the decomposition of the material [25, 26, 27]. Other 2D materials were likewise investigated for their protective use, and it could be demonstrated that covering BP with monolayer graphene can prolong the lifetime of the BP nanosheets [28, 29, 30]. While effective for passivation, a common drawback of these methodologies is that they prevent subsequent surface modifications required for specific applications.

Covalent functionalization of BP is another attractive and highly effective strategy for the protection of BP, as it utilizes the unpaired electrons on each phosphorus atom to form new covalent bonds, reducing BP's tendency to react with surrounding oxygen species. This drastically prolongs the lifetime of BP nanosheets, extending it from mere hours after exfoliation to over 200 days for covalently functionalized BP with aryl diazonium salts [31]. Beyond diazonium salts, various other reagents for the covalent functionalization of BP are known, including nucleophilic reagents such as alkyl halides [32, 33], amphiphobic reagents [34], aryl iodonium salts [35], azides [36], through surface coordination [37, 38], or by using further radical‐generating reagents such as AIBN [39]. Most of the developed procedures for the covalent functionalization of BP were adapted from graphene research and proved to be feasible after slight adjustments, offering the opportunity to modify and adjust surface properties on demand and allowing for tailor‐made modifications, which are especially important for certain applications [35, 40].

Lately, in the case of graphene, the concept of precisely controlling the covalent functionalization of 2D materials has been extended toward fine‐tuning their intrinsic properties in a spatially defined manner. This approach allows for high control of the functionalization process and enables the creation of well‐resolved patterns near the Abbé limit by activating an adsorbed reactive precursor compound through laser irradiation. A significant advantage of such a laser‐driven addend binding is that the geometry and shape of the covalently functionalized regions of the respective 2D material can be arbitrarily chosen, being solely tied to the laser pathway. This method, often referred to as laser patterning, involves the photo‐induced activation of a suitable precursor, generating radical species. These subsequently react with the graphene surface, leading to covalent addend‐binding and enabling a mask‐less, direct writing of chemical information [41, 42, 43, 44].

Building on these advances, our group has successfully implemented dibenzoyl peroxides (DBPOs) as versatile precursor reagents for such laser‐triggered covalent functionalization of graphene [43, 45]. Here, graphene acts as a photosensitizer, and upon laser irradiation, an electron is injected from the extended *π*‐system of graphene into the lowest unoccupied molecular orbital (LUMO) of the adsorbed DBPO species. The DBPO molecules subsequently decompose to form reactive phenyl radicals that attack the graphene lattice, thereby introducing sp^3^‐hybridized lattice carbon atoms due to the attachment of functional moieties [46]. Beyond single‐layer graphene, this concept has been successfully utilized for the synthesis of covalently linked heterostructure assemblies. Here, CVD‐grown molybdenum disulfide (MoS_2_) was grafted to a monolayer graphene sheet by laser‐activation of precursor aryl units that were introduced to the MoS_2_ via covalent functionalization prior to the heterostructure formation. These functional aryl moieties were strategically placed between the two different 2D material layers [47], allowing for the precise manipulation of the intrinsic properties of both materials, thereby leading to refined heterostructures.

With respect to BP, similar approaches have been investigated for the covalent “patterning” of few‐layer BP systems; however, most reports primarily focused on the selective and locally‐induced oxidation of BP through either photo‐oxidation [48] or by scanning probe nanolithography [49]. A spatially‐controlled introduction of new P─C bonds using a laser‐triggered covalent functionalization procedure remains unexplored to date.

Herein, we present a detailed analysis of the stability of BP nanosheets under laser irradiation in the context of the laser‐triggered, covalent functionalization approach. More precisely, we evaluated the fundamental feasibility of the photo‐induced, covalent functionalization of pristine BP nanosheets using *bis*(4‐chlorobenzoyl)peroxide (Cl‐DBPO) as the radical precursor, following the laser patterning approach established by Edelthalhammer et al. for the 2D engineering of graphene [43]. Furthermore, this procedure was adapted for a graphene‐BP (G‐BP) heterostructure assembly, which was assembled using a step‐by‐step dry transfer technique, and subsequently, top‐side functionalization of the superjacent monolayer graphene utilizing Cl‐DBPO as a model compound was successfully achieved. Our findings confirm that further chemical modifications via laser patterning are indeed possible on these systems, setting up a crucial first step toward versatile BP‐based hybrid systems with advanced functionality and tunability.

## 2 Results and Discussion

While grafting chemical functionalities onto 2D materials using the laser writing concept has been successfully applied for the locally‐controlled covalent patterning of graphene, its adaptation to BP requires a comprehensive understanding of how and to what extent laser irradiation inherently affects the BP nanosheets. Previous reports have shown that laser‐induced photo‐oxidation of BP is possible and that the level of oxidation depends on the applied laser power [48]. Consequently, a systematic investigation into the behavior of few‐layer BP nanosheets under laser irradiation was conducted, which exposed mechanically exfoliated BP nanosheets to laser light with varying parameters, including the laser power and irradiation time.

**FIGURE 1:**
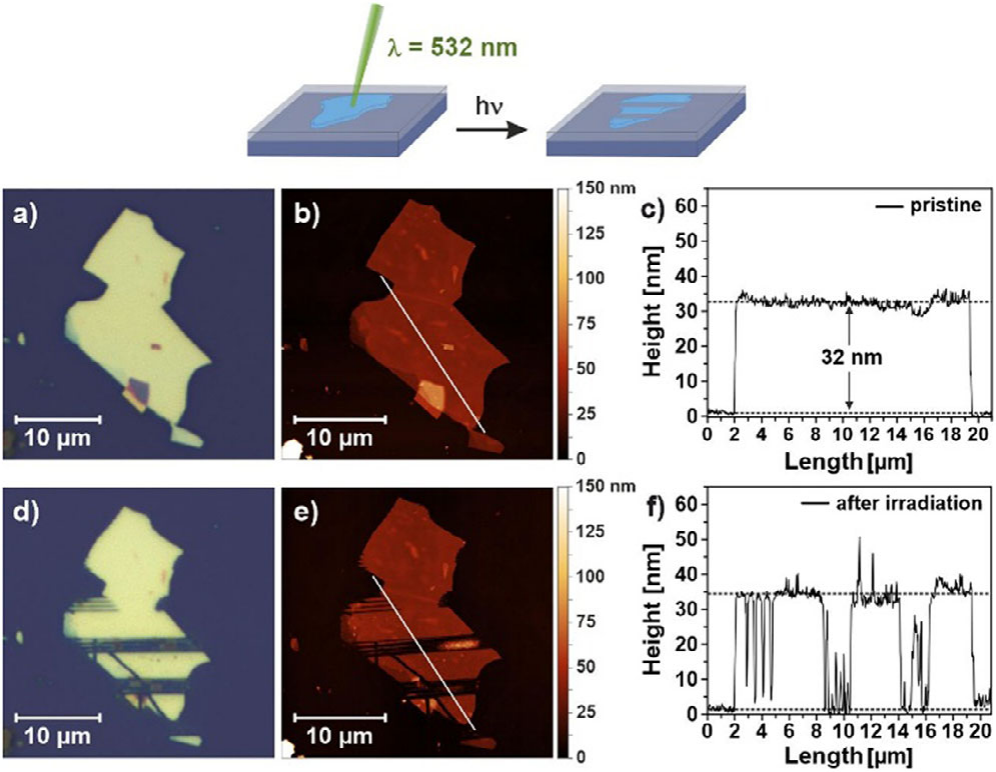
Characterization and comparison of a laser‐irradiated BP flake to its pristine counterpart. Optical (a) and AFM images (b) of a few‐layer BP flake prior to the laser irradiation confirm its pristine nature. A corresponding height profile (c) extracted across the white line marked in (b) reveals its initial thickness. Optical (d) and AFM images (e) of the identical BP flake after locally‐controlled laser irradiation. Horizontal rectangular regions of the BP nanosheet were exposed to a 532 nm laser with varying laser powers (see Supporting Information for detailed experimental parameters). For comparison, a second height profile was extracted after the laser exposure (f) from the same position indicated in (e), illustrating the changes in topography after the laser exposure.

BP is commercially available as bulk crystalsand needs to be exfoliated before use to enable access to its surface chemistry. Few‐layer BP can be obtained through mechanical exfoliation of the bulk material (Supporting Information) [50]. Moderately thin flakes with a thickness of around 32 nm were irradiated under ambient conditions using a green laser (*λ*  =  532 nm) mounted onto a WITec alpha300 R confocal Raman setup (Figure 1). Keeping the irradiation time constant at 3 s, the applied laser power was systematically varied between 1 and 22 mW (Figure S1). To assess structural changes of the BP nanosheets depending on these parameters, Raman area mappings in the shape of rectangular patterns were performed. For each line pattern, a different laser power was applied, selectively exposing distinct regions of the BP surface to the laser irradiation.

**FIGURE 2:**
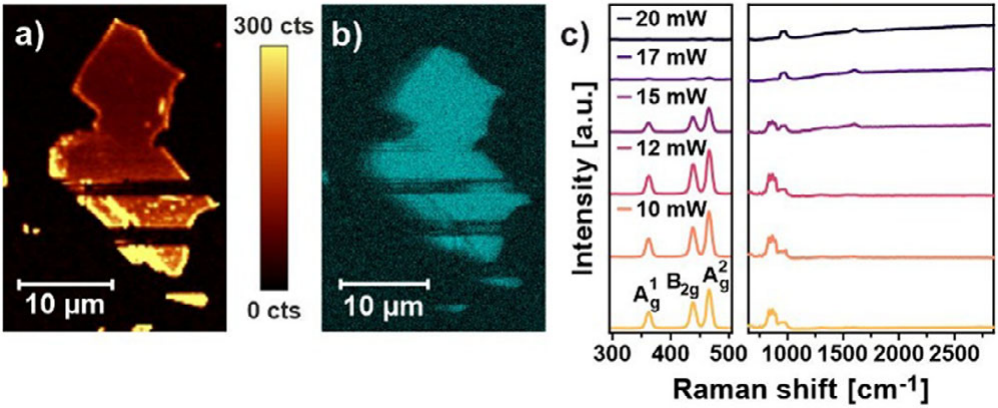
Raman and EDS characterization of the irradiated BP flake is shown in Figure 1. After the laser exposure, a Raman map illustrating the *A^1^_g_* band of BP (a) and an EDS mapping displaying the elemental distribution of phosphorus (b) were recorded, and additionally, the corresponding mean Raman spectra extracted from each of the irradiated areas, compared to the non‐irradiated parts of the BP flake, are shown in (c). Spectra were vertically offset, and the three characteristic bands of BP, the *A^1^_g_* band, the *B_2g_* band, and the *A^2^_g_* band (in this order from low to high wavenumbers) are indicated in the non‐irradiated BP spectrum at the bottom.

Starting at 15 mW, the laser irradiation visibly alters the pristine BP flake, creating sharp, seemingly “burnt” lines (Figure 1d). This trend becomes more pronounced with increasing laser power. These lines appear as dark regions under the optical microscope, resembling the underlying substrate rather than the original BP flake. Atomic force microscopy (AFM) images of the same BP flake, taken before and after irradiation, corroborate these observations (Figure 1b,e). Initially, freshly prepared BP nanosheets exhibit a homogeneous and even surface, allowing for a facile determination of the flake height, which in this case was approximately 32 nm. After irradiation, deep trenches or cuts are evident in the BP structure, and the etched depth directly depends on the applied irradiation parameters. At high laser powers, the irradiated regions of the flake were ablated, resulting in a depth of roughly 32 nm according to the corresponding AFM height profile, indicating the complete removal of the BP material down to the substrate (Figure 1f). To further characterize these changes, Raman spectroscopy and energy‐dispersive x‐ray spectroscopy (EDS) mappings were performed on the same BP flake after the laser irradiation (Figure 2).

These measurements confirmed that in the laser‐irradiated areas with powers exceeding 15 mW, no spectral or elemental evidence for the presence of crystalline BP could be found, directly correlating with the dark lines observed in the corresponding mappings (Figure 2a,b). On the other hand, EDS mappings did not reveal a significant increase in the oxygen content within these irradiated regions (see Figure S2, for full EDS characterization). However, this was to be expected given the previously observed complete ablation of the nanosheet structure in these areas by AFM, which would leave no phosphorus and therefore likewise no phosphorus oxide‐containing residues for detection. Nonetheless, bubble‐like residues were observed in the pattern irradiated with 17 and 20 mW, which resemble liquid phosphonic and phosphoric acid bubbles, which are known to form when BP nanosheets are exposed to ambient conditions [51, 52]. Despite the absence of characteristic Raman signals for pristine BP, these residues still exhibit weak elemental phosphorus signals and a brighter oxygen contrast in the EDS mappings. This strongly suggests that, as also observed by Lu et al. [48], laser‐induced oxidation is the primary mechanism responsible for the degradation of the BP flake in these regions. Irradiation with lower laser powers (below 15 mW), on the other hand, did not induce any observable changes (Figure S3).

Similar to many other layered 2D materials, BP shows a highly thickness‐dependent behavior, which can, for instance, be seen in its tunable bandgap according to the number of layers of the BP nanosheet [53]. Therefore, we likewise investigated the behavior of thin (around 8–10 nm) BP flakes under laser irradiation (Figure S4). Identical alterations and trends could be observed, although the laser power threshold upon which photo‐oxidation occurs was significantly lower than for the presented thicker BP nanosheets. Instead of showing signs of material ablation at around 15 mW, the thinner BP nanosheets were severely damaged by the laser irradiation starting at around 7 mW. These findings correlate well with previous reports, showing that thinner BP flakes are more prone to oxidative degradation under ambient conditions and are therefore less stable in general [23].

**FIGURE 3:**
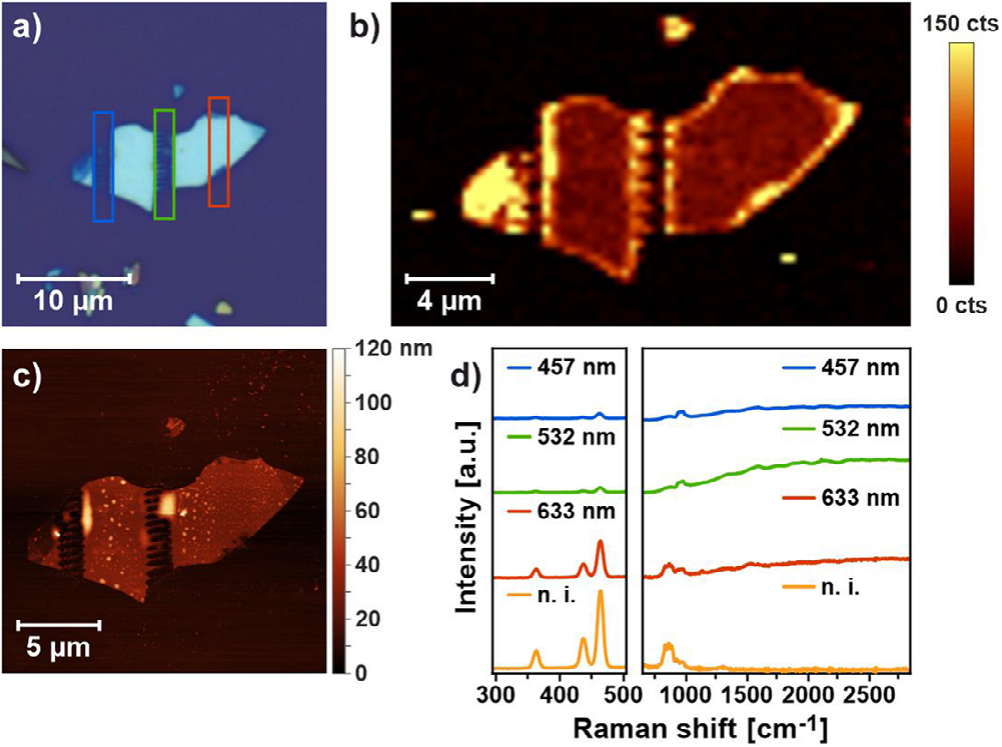
Characterization of a BP flake after being irradiated with three different laser wavelengths at 15 mW and 3 s. The optical image after irradiation (a) exhibits partially visible lines in the marked squares that were irradiated with a 457, 532, and a 633 nm laser (from left to right). Additionally, a Raman mapping was conducted after irradiation (b), where the two irradiated areas on the left and the middle of the flake show decreased intensities of the *A^1^_g_* band of BP. Similar trends are observable in the extracted mean Raman spectra in (d), as the intensities of all BP bands decrease and background fluorescence appears with lower wavelength irradiation. Spectra were vertically offset. The spectrum of the non‐irradiated parts of the BP flake is indicated with n.i. for comparisons, and the corresponding AFM image of the BP flake after irradiation is shown in (c).

To gain a deeper insight into this behavior, the influence of the laser wavelength on the BP flake alteration was further investigated using three different lasers equipped to our Raman setup, irradiating one BP nanosheet with a blue (457 nm), green (532 nm), and red (633 nm) laser. For these experiments, solely the wavelength of the laser irradiation was varied, while the laser power and the irradiation time were kept constant at 15 mW and 3 s (Figure 3).

Applying the same set of parameters while varying the laser wavelength of the laser light irradiated on one mechanically exfoliated BP flake revealed a distinct dependency of the observed alterations on the incident photon energy. Both the blue (2.71 eV) and the green laser (2.33 eV) supply sufficient energy to the system to initiate degradation and most likely concomitantly oxidation of the observed BP flake. In contrast, the red laser (1.96 eV) was not able to introduce such prominent changes, and only a slight thinning of the more reactive rims of the BP flake can therefore be observed (Figure S5). This aligns well with observations about the photo‐degradation of BP, where it was shown that lower wavelength irradiation leads to faster oxidation of the BP nanosheets [54].

Building on our fundamental understanding of BP nanosheets under laser irradiation, we next investigated the possibility of a laser‐triggered covalent functionalization of BP. This was pursued in analogy to well‐established protocols for graphene, employing DBPO‐based precursor compounds [43, 45]. Specifically, we utilized *bis*(4‐chlorobenzoyl)peroxide (Cl‐DBPO) for the laser‐triggered covalent functionalization procedure, as the incorporated chlorine atoms allow for further characterization of the obtained results by elemental analysis.

**SCHEME 1:**
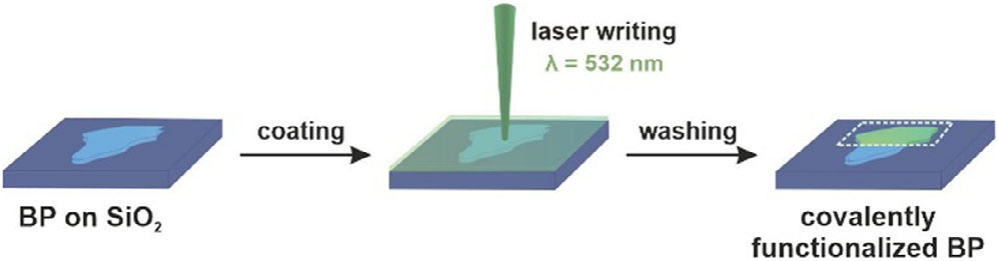
Schematic representation of the attempted covalent functionalization procedure of BP using Cl‐DBPO.

As illustrated in Scheme 1, samples with mechanically exfoliated BP nanosheets, transferred to Si/SiO_2_ wafers, were prepared and subsequently spin‐coated with one droplet of a Cl‐DBPO solution (c = 1.6×10^−2^ mol/L in diethyl ether). Subsequently, suitable BP flakes were identified for the laser irradiation.

After selecting a suitable BP flake, half of the flake covered with the Cl‐DBPO coating was irradiated with a green laser (*λ*  =  532 nm). Parameters for the laser irradiation were carefully selected according to our preceding study concerning the irradiation of pristine BP nanosheets. To prevent laser‐induced photo‐oxidation of the material, only very low laser powers of 0.5 mW and short irradiation times of 0.1 s were selected for the direct laser writing approach. It could be shown that in the case of graphene, such mild laser writing conditions are more than sufficient to graft a notable degree of functional moieties onto the graphene lattice [45], rendering them ideal for the laser‐triggered covalent functionalization of BP. Subsequently, the residual coating was removed in a washing step to prevent further functionalization of the sample upon the laser‐based characterization. Irradiating solely one half of the investigated BP flakes offers an internal reference, allowing for a direct comparison between irradiated and non‐irradiated areas of the BP nanosheets.

**FIGURE 4:**
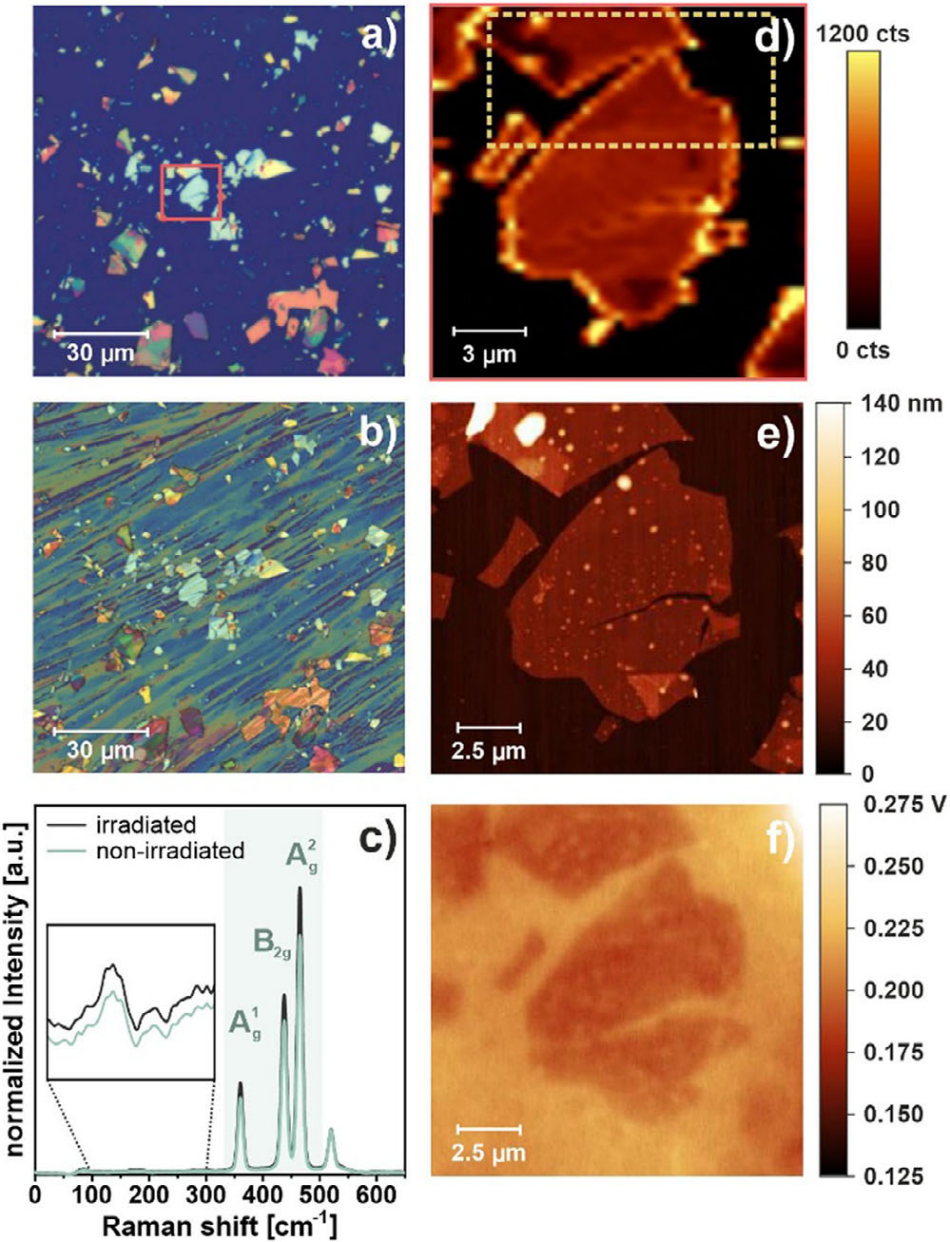
Characterization of the direct laser writing attempt of BP. Optical images of (a) the pristine wafer after mechanical exfoliation, highlighting the selected flake for the laser writing, and (b) after spin‐coating with Cl‐DBPO solution (c = 1.6×10^−2^ mol/L). (c) Mean Raman spectra were extracted from the Raman map shown in (d), which illustrates the intensity distribution of the *A^1^_g_* band of BP. The irradiated area of the presented BP nanosheet is marked in orange and was irradiated with a laser power of 0.5 mW and an integration time of 0.1 s. (e) and (f) illustrate the topography and the surface potential of the BP flake from (d).

A detailed analysis of the Cl‐DBPO‐coated BP sample, measured and recorded after the laser irradiation and washing step, is presented in Figure 4. Optical images before and after coating the sample illustrate the BP flake selection by optical contrast and the coating homogeneity (Figure 4a,b). Although the selected BP flake is entirely covered with the Cl‐DBPO coating, the coating thickness is not homogeneous over an extended area. More detailed insights into the topography of the flake were obtained from AFM measurements. The observed flake shows incipient signs of oxidation due to exposure to ambient conditions, evident by the formation of nanobubbles on the surface (Figure 4e) [22]. The overall flake, however, remains intact and reveals a homogeneous thickness across the even surface.

The corresponding Kelvin probe force microscopy (KPFM) measurement, providing insights into the local surface potential of the scanned area, reveals a surface potential drop in the substrate regions with transferred BP nanosheets, which is to be expected due to the different chemical nature of the substrate compared to BP. However, when comparing the irradiated area of BP with the non‐irradiated one, no apparent differences in surface potential can be discerned (Figure 4f).

Furthermore, mean Raman spectra recorded in the irradiated and non‐irradiated regions of the flake do not show any signals above 600 cm^−1^ and even a close‐up into the low wavenumber region does not reveal the emergence of new spectral bands that would indicate either the covalent functionalization of BP or the breaking of existing P─P bonds (Figure 4c). To gain more insights into the elemental composition of a given area, an EDS measurement was conducted. However, the measurement did not reveal a higher carbon content or the accumulation of chlorine in the irradiated areas of the BP flake (Figure S6), indicating that no covalent functionalization of the pristine BP nanosheets occurred. Further coating techniques and solvents were tested in order to evaluate a possible influence of these parameters on the outcome of the attempted laser writing; however, no further evidence for a successful covalent functionalization of the investigated BP nanosheets was observed (see Supporting Information for further experimental details).

Therefore, the material system itself was optimized to investigate if the stability of BP nanosheets under laser irradiation can be improved. As previously discussed, other 2D materials can be utilized as a protective “coating” layer for BP and it could be shown that covering BP with monolayer graphene can prolong its lifetime [29]. Building on these findings, it seems promising to enhance the stability of BP nanosheets against laser irradiation in a similar fashion, where the superjacent graphene layer impedes the penetration of reactive oxygen species responsible for the oxidative degradation of BP. Besides, investigating a more controllable system that allows for a straightforward characterization of the attempted laser‐initiated, covalent functionalization facilitates further investigations.

This approach raises two key questions. First, since the laser‐triggered functionalization verifiably works on graphene, would it likewise be possible to covalently attach functional moieties to the adjacent graphene layer when a van der Waals (vdW) heterostructure with BP is formed? And second, would the top‐side functionalization of graphene trigger a locally‐defined backbonding to the underlying BP nanosheets and establish an antaratopic bonding? These two questions are directly related, as the covalent attachment of functional groups from one side of the extended graphene lattice introduces strain to the system due to the unilateral pyramidalization of lattice carbon atoms upon rehybridization. Via antaratopic covalent backbonding to the underlying substrate, this built‐up strain can, in principle, be released [55, 56] and in the case of a G‐BP heterostructure, a successful backbonding could therefore potentially promote the formation of interlayer phosphorus─carbon bonds between both materials.

**SCHEME 2:**
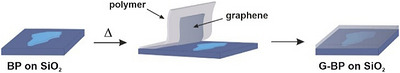
Schematic representation of the dry transfer technique to assemble the G‐BP heterostructure.

To investigate this in detail, a vdW‐based G‐BP heterostructure was assembled by combining mechanically exfoliated BP nanosheets with CVD‐grown graphene using a step‐by‐step dry transfer technique (Scheme 2).

**FIGURE 5:**
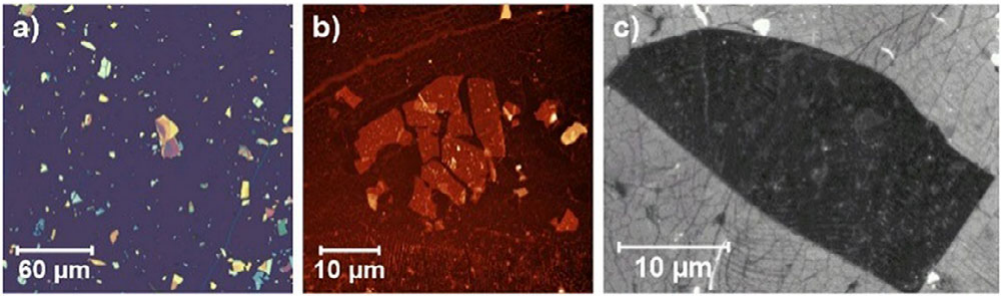
Formation of the G‐BP heterostructure. Optical image (a), AFM image (b), and an SEM image (c) illustrate the optical appearance of multiple vdW‐mediated hybrid systems between CVD‐grown graphene and BP nanosheets.

At first, BP crystals were mechanically exfoliated and transferred to Si/SiO_2_ wafers inside an argon‐filled glovebox and were pre‐heated to 115°C. Subsequently, CVD‐grown graphene was transferred onto a polymer sheet, cut into pieces, and transferred to inert conditions. Each piece of graphene on the polymer was then pressed onto the pre‐heated, BP‐covered wafers and left on the hot plate for 40 min to ensure the transfer of heat to the transparent polymer carrying the graphene. The applied heat increases the plasticity of the polymer, reducing the adhesion to the graphene sheet and allowing a facile transfer of the 2D material to a new substrate. The protective PMMA layer was thereupon removed, yielding the G‐BP heterostructure. Optical images illustrate the various types of BP nanosheets that were transferred upon their mechanical exfoliation by their varying color contrast. The superjacent graphene sheet on top, on the other hand, is nearly invisible but still fully intact (Figure 5). More precise optical measurements using an AFM or SEM reveal two key structural features: the well‐defined shapes and lateral dimensions of the BP nanosheets, and the presence of small wrinkles or holes in the graphene sheet. The latter, observed even when directly on top of the BP nanosheets, confirms the formation of a vdW‐based heterostructure between the two 2D materials.

**FIGURE 6:**
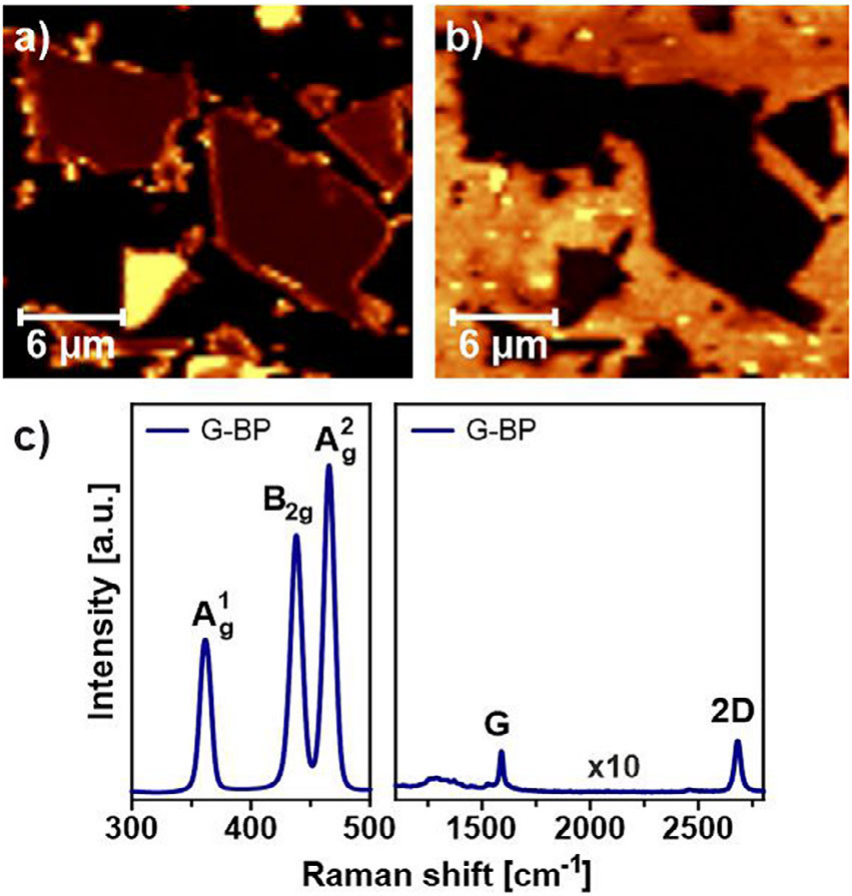
Raman characterization of the G‐BP heterostructure. Raman mappings illustrating the *A^1^_g_* band intensity of BP (a) and the G band intensity of graphene (b). The respective mean Raman spectrum, extracted from the areas of the G‐BP heterostructure, is shown in (c), and all visible Raman bands of BP and graphene are indicated.

Raman measurements (Figure 6) clearly confirm the presence of both materials in the heterostructure assembly. The three characteristic BP bands are simultaneously observed with the *G* and *2D* mode of graphene in a single area. A Raman mapping covering the region of BP nanosheets and their immediate surroundings confirm that the BP flakes can be readily identified when imaging the characteristic *A^1^_g_* mode of BP. Interestingly, while the *G* and *2D* band intensities are distributed over the entire measured area, both are diminished in the regions covering the BP nanosheet. This leads to a dark “shadow” of the flakes when imaging the graphene Raman bands. To the best of our knowledge, such a behavior has not yet been described in literature [57, 58, 59], although Raman spectra of G‐BP heterostructures zoomed into the spectral region of the graphene modes were shown [60], suggesting a similar phenomenon may have been observed.

**FIGURE 7:**
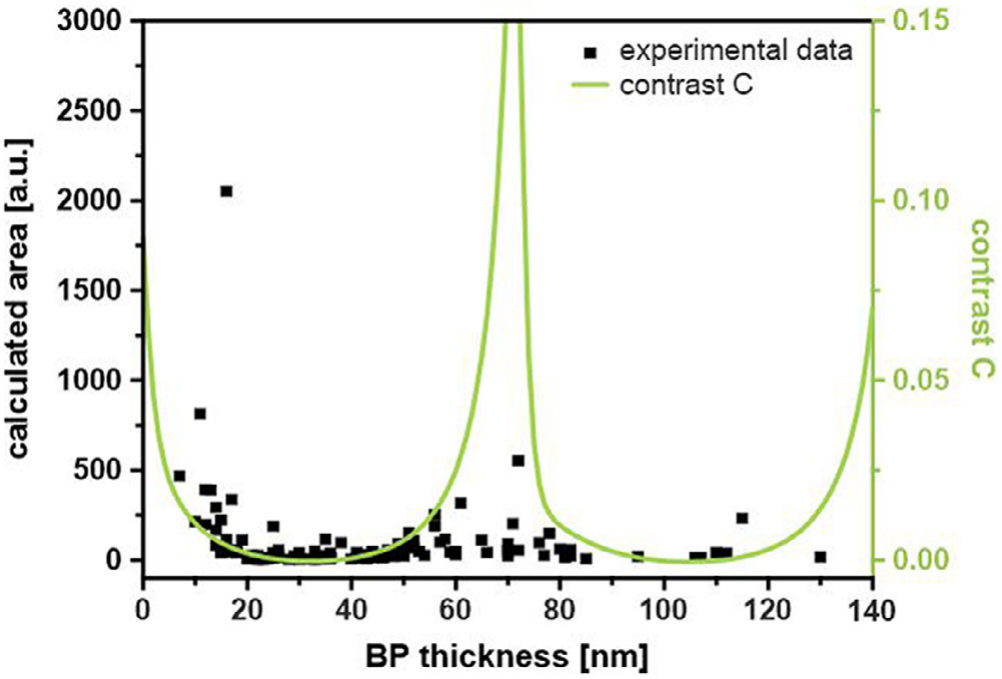
Comparison between the simulated contrast C and experimental data obtained from the G‐BP heterostructures. The black dots represent calculated area values after fitting the G band of various single Raman spectra, where each dot equals one value from a measured G‐BP hybrid. The height determination of the BP nanosheets was conducted with AFM topography measurements. The green line represents the simulated contrast C of the four‐interface system derived from Equation S2.

For a deeper understanding of the intensity decrease of the graphene Raman modes, we performed intensity simulations based on interference effects in the present four‐interface system consisting of air, graphene, BP, SiO_2_, and silicon. Details of the simulation, including the governing equation (Equation S1) and optical parameters, are provided in the Supporting Information. Figure 7 compares the experimental results, obtained from a correlation between single point Raman spectra of the G‐BP hybrids and the corresponding height of the BP flakes, with the simulated reflectance contrast for the graphene *G* mode as a function of the BP nanosheet thickness when excited at *λ* = 532 nm. The black dots represent the experimentally obtained *G* mode intensities as a function of the thickness of the underlying BP nanosheet, while the green line represents the simulated intensity dependence. To evaluate the reflected signal associated with the graphene layer, the simulated curve shows the contrast C (Equation S2), as the relative change in reflectance between the BP/SiO_2_/Si substrate with and without a superjacent graphene layer. Overall, the simulated intensities of the graphene Raman modes are in good agreement with the experimental data (Figure 7), and the reduced graphene signal observed for BP thicknesses in the range of 20–50 nm is accurately reproduced by the simulated interference effects of the four‐interface system. At certain BP thicknesses, destructive interference strongly suppresses the reflected signal, leading to a minimal or undetectable graphene *G*‐mode intensity. Conversely, the model predicts an enhanced graphene signal for BP thicknesses around 50–80 nm, which is partially corroborated by the experimental data.

The established correlation between the BP nanosheet thickness and the graphene *G* mode intensity of the respective G‐BP heterostructure, hence allows for the efficient optical selection of G‐BP hybrids to investigate their laser‐triggered, covalent functionalization in detail. Below a BP nanosheet thickness of 20 nm, the reduction of the graphene signal due to interference effects is only minimal, enabling a full characterization of the heterostructure system via Raman spectroscopy.

**FIGURE 8:**
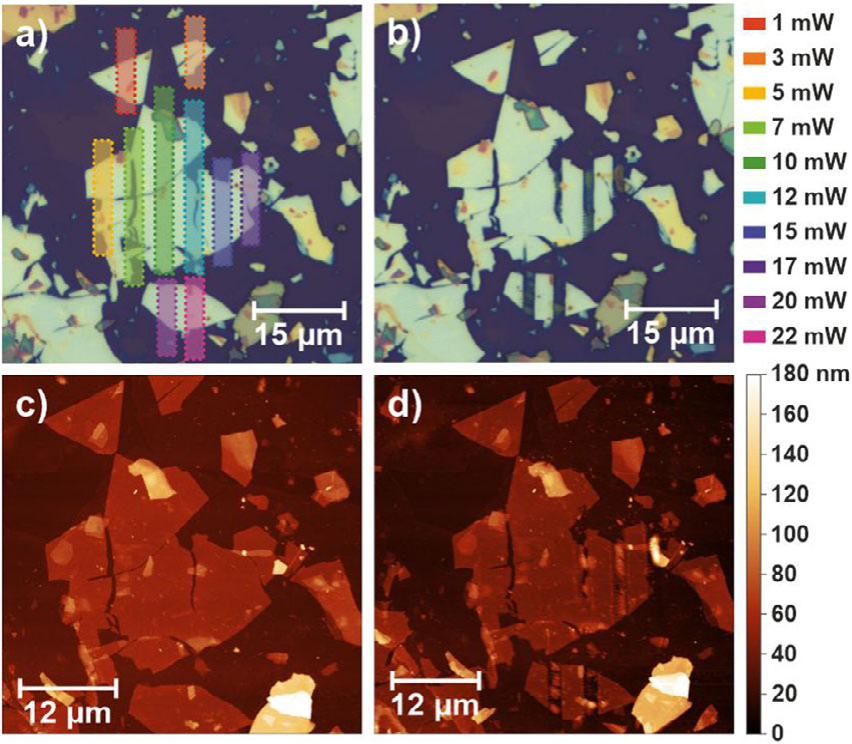
Photo‐oxidation of G‐BP heterostructures. BP nanosheets covered with graphene were exposed to various laser irradiation conditions using a green laser (*λ* = 532 nm), where the laser power was varied between 1 and 22 mW and the irradiation time was kept constant at 3 s. Optical and AFM images before (a) and (c), and after irradiation (b) and (d) show alterations of the BP nanosheet structure with high laser power irradiation.

As mentioned above, laser patterning is a facile and straightforward method to introduce functional moieties to the graphene lattice under relatively mild laser irradiation conditions. Since locally‐induced, covalent functionalization of such G‐BP heterostructure systems has not been explored, we transferred the laser writing concept established for graphene to our presented G‐BP hybrids. For that purpose, an optimized protocol developed in our group [45] was utilized, and Cl‐DBPO was employed as the reactive reagent [61]. In analogy to Figure 1, first, a systematic study of the laser irradiation conditions was conducted on the G‐BP heterostructure to test the system's stability and its limits (Figure 8). Using the same rationale, a BP nanosheet with a thickness of 26 nm, covered with graphene, was selected and exposed to laser irradiation (*λ*  =  532 nm). A constant irradiation time of 3 s was used, whereas the laser power was varied from 1 to 22 mW, the maximum laser output of our setup. To ensure identical conditions, one spot with BP nanosheets of mostly uniform thickness was selected, and solely a small rectangular part of the BP nanosheet was irradiated with one set of parameters (Figure 8a). Starting with a laser power of 12 mW, alterations in the BP structure could be observed, both visible under the optical microscope and when recording an AFM image of the respective BP flake.

Similar to our previous observations on bare BP, deep trenches appeared, and more of the pristine material seems to be ablated with increasing laser power. This suggests that, although graphene can prolong the lifetime of BP nanosheets, it cannot fully prevent the laser‐induced photo‐oxidation. The slightly lower threshold for the ablation of BP compared to Figure 1 can be attributed to the lower thickness of the investigated BP nanosheets. These findings provide valuable insights for the subsequent parameter selection concerning the covalent functionalization of the G‐BP heterostructure via laser patterning.

**SCHEME 3:**
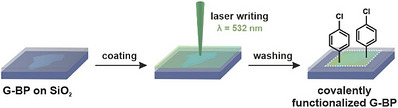
Schematic representation of the covalent functionalization of the G‐BP heterostructure using the laser patterning technique.

Following the investigation into the behavior of the G‐BP heterostructure under laser irradiation, the laser‐triggered, covalent functionalization of such hetero‐assemblies was explored. Therefore, G‐BP heterostructures were prepared as described above by transferring CVD‐grown graphene onto mechanically exfoliated BP nanosheets using a stamping technique. After coating the sample by drop‐casting a Cl‐DBPO solution (c = 10^−3^ mol/L in THF), a laser writing was performed on the G‐BP heterostructure under commonly used graphene patterning conditions (Scheme 3).

**FIGURE 9:**
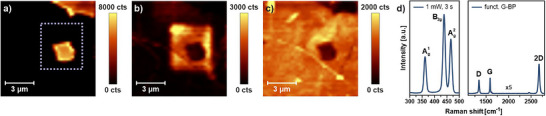
Covalent functionalization of G‐BP via laser writing using a 532 nm laser, 1 mW laser power, and 3 s integration time. Raman maps, illustrating the *A^1^_g_* band of BP (a), the *D* band (b), and the *G* band (c) of graphene, were recorded after the laser patterning procedure. A corresponding mean Raman spectrum of the small G‐BP heterostructure area is shown in (d), whereas the intensity of the graphene bands was increased five times for visibility, and all respective Raman bands of BP and functionalized graphene are indicated.

A sample with G‐BP hybrids, featuring rather thin BP nanosheets, was prepared. After coating the wafer with a Cl‐DBPO solution, a rectangular area around one of the BP flakes was irradiated with a 532 nm laser, while using only very low laser powers to avoid photo‐oxidation. Therefore, a laser power of 1 mW and an integration time of 3 s were selected. After irradiation, the residual coating was washed away, and a Raman read‐out was performed to investigate spectral changes at the same spot (Figure 9). A Raman map illustrating the *A^1^_g_* band intensity of BP clearly shows the mechanically exfoliated BP nanosheet. When imaging the intensity distribution of the *D* and *G* bands over the measured area, further information can be obtained. Although graphene covers the entire measured area, a clear *D* band emerged only in the laser‐irradiated region. This confirms the locally‐controlled introduction of sp^3^ carbon atoms into the graphene lattice exclusively in the laser‐exposed regions. Additionally, in both Raman mappings illustrating the intensity distribution of graphene‐related Raman bands (Figure 9b,c), the previously described decline in signal intensity can be clearly observed. These qualitative observations are confirmed by extracting a mean Raman spectrum of the functionalized G‐BP heterostructure. The three characteristic Raman bands of BP are present, as well as three graphene‐related bands. In addition to the *G* and *2D* band, a visible *D* band emerged, and even a small *D’* band is likewise apparent as a shoulder on the right side of the *G* band. These spectral characteristics all confirm the successful covalent attachment of functional moieties to the graphene layer and have not been observed during the local irradiation performed in Figure 8 under the same laser writing parameters (Figure S7).

As previously mentioned, light irradiation causes photo‐oxidation of the material [23, 62], leading to the partwise or complete degradation of BP by laser irradiation at extensive laser powers. To differentiate between photo‐oxidation and covalent functionalization, the *A^1^_g_/A^2^_g_* ratio calculated from the obtained Raman spectra can be used. This ratio is highly sensitive to the degree of oxidation of the observed flake, as pristine BP nanosheets statistically show a ratio of 0.4–0.6, and these values drastically decrease upon oxidation [62]. From the recorded Raman spectra (Figures S8 and 9d), an *A^1^_g_/A^2^_g_* ratio before the laser treatment of 0.72 can be calculated and afterwards the ratio only slightly drops to 0.66, confirming that the sample was not entirely oxidized even after laser treatment. Nevertheless, this decrease indicates an increase in surface oxides, most likely due to the prolonged exposure to ambient conditions during the sample processing.

**FIGURE 10:**
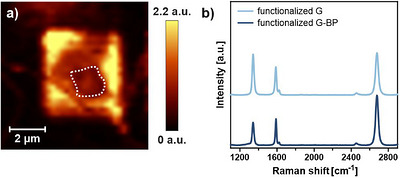
Comparison between the degree of functionalization on graphene and the G‐BP heterostructure in the dotted area after laser writing with Cl‐DBPO. Raman map illustrating the intensity distribution of the I_D_/I_G_ ratio (a) and the corresponding mean Raman spectra extracted solely from the G‐BP heterostructure region compared to the surrounding graphene area are shown (b).

To compare the extent of functionalization on graphene and the regions of the G‐BP heterostructure, changes in their mean Raman spectra were analyzed. The ratio between the *D* band intensity and the *G* band intensity (*I_D_/I_G_* ratio) is a widely used metric to ‘quantify’ the degree of functionalization of various graphene samples. A higher *I_D_/I_G_* ratio indicates a higher degree of functionalization when the average distance between defects does not fall below 3–4 nm. This direct correlation between the quantity of defects in graphene and the calculated *I_D_/I_G_* ratio was established by Cançado [63] and Lucchese [64], and remains valid under the abovementioned condition. Analyzing the mean Raman spectra of functionalized graphene and the functionalized G‐BP heterostructure area shown in Figure 10a, clear differences can be discerned. The functionalized G‐BP heterostructure exhibits an *I_D_/I_G_* ratio of approximately 0.8, while the functionalized graphene supported on Si/SiO_2_ shows a significantly higher ratio of approximately 1.2 when irradiated with identical parameters (Figure 10b), indicating that the covalent functionalization of graphene is less efficient when the material is in contact with BP.

Previous reports have demonstrated that the substrate plays a crucial role in the covalent functionalization of graphene [55, 65], irrespective of whether a support material or a second 2D material was used. Efficient backbonding is required to release strain associated with the supratopic functionalization, especially when high quantities of functional moieties are grafted to the graphene lattice [66]. For instance, a lower degree of functionalization was achieved on graphene supported by hexagonal boron nitride (hBN) compared to graphene deposited on Si/SiO_2_ when using identical functionalization parameters [67]. This suggests that, similar to hBN, BP nanosheets are less effective for the required backbonding than SiO_2_.

**FIGURE 11:**
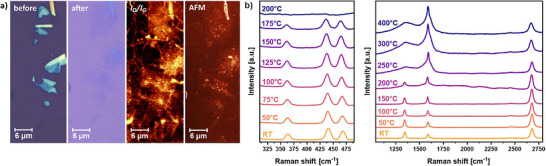
Characterization of the functionalized G‐BP heterostructure and the surrounding graphene after T‐dependent Raman measurements. The four images show the course of the sample before and after heating (a). The corresponding I_D_/I_G_ Raman map and an AFM image of the same spot are likewise shown in (a) for the same sample after allowing it to cool down to RT. The respective mean Raman spectra of the functionalized region (G‐BP heterostructure and the surrounding graphene) are shown in Figure 9b, measured at the marked temperature, and are divided in the spectral region of the BP bands (left) and the graphene Raman modes (right). All spectra in (b) were normalized to the first Si Raman band for comparison.

Depending on the type of functional moieties that are introduced, heat‐induced defunctionalization was proven to be feasible to re‐obtain pristine graphene, especially for molecules with simpler binding motifs [43, 68, 69]. To verify the reversibility of the investigated covalent functionalization of the G‐BP heterostructures, a temperature‐dependent (T‐dependent) Raman experiment was conducted, where a functionalized sample was heated stepwise from room temperature (RT) to 400°C. Below 150°C, no apparent changes of the BP signals can be seen, at higher temperatures, the three characteristic bands steadily decrease in intensity, until they disappear at 200°C. As the BP signals vanish, the background of the Raman spectrum significantly increases, and all graphene modes broaden. These spectral changes, which show no further evolution up to 400°C, suggest that an irreversible reaction took place (Figure 11b). A plausible explanation could be that graphene reacts with reactive decomposition fragments of the underlying BP nanosheets. Since the graphene layer on top should trap these fragments, they are unable to evaporate or diffuse, leading to the establishment of additional defects in the graphene lattice. As this process would most likely be uncontrolled and highly dependent on the chemical nature of the decomposition fragments, it would account for the observed increase in the *D* band intensity and the signal broadening, despite the expected detachment of functional moieties [43].

An analysis of a large‐area Raman map acquired after the T‐dependent Raman experiment reveals that the spectral features indicating an irreversible change in the graphene structure are exclusively recorded in areas where previously BP nanosheets were located and in their direct vicinity. The complete decomposition of the BP flakes is further confirmed by an AFM topography image (Figure 11a, right). Here, only bubble‐like, undefined structures consistent with previous reports of BP oxidation [70] are visible. Nonetheless, structural features of the graphene layer, such as wrinkles, remain visible even above these nanobubbles, indicating that the graphene sheet remains intact on a microscopic level. This finding supports the hypothesis that the graphene sheet acts as a physical barrier trapping the decomposition fragments of the underlying BP nanosheets, leading to the irreversible reaction observed in the Raman spectra (Figure 11b).

## 3 Conclusion

In conclusion, we have presented a thorough investigation of the laser‐induced, covalent functionalization of BP nanosheets and G‐BP vdW heterostructures using dibenzoyl peroxide‐based precursors. We could show that mechanically exfoliated BP nanosheets exhibit high sensitivity to laser irradiation under ambient conditions, with complete ablation of the material occurring above 15 mW (*λ*  =  532 nm) through photo‐induced oxidation. This degradation shows strong wavelength and thickness dependency, with thinner flakes degrading at lower laser power thresholds. Direct, laser‐triggered functionalization of pristine BP using *bis*(4‐chlorobenzoyl)peroxide yielded no evidence of successful covalent attachment of functional moieties, indicating that graphene functionalization protocols cannot be directly transferred to BP. To overcome this limitation, G‐BP heterostructures were assembled via a dry transfer of CVD‐grown graphene onto BP. Raman spectroscopy of the assembled G‐BP heterostructures revealed an unexpected phenomenon. The characteristic *G* and *2D* band intensities of graphene were significantly diminished in regions covering the BP nanosheets. Our simulations demonstrated an excellent agreement with the experimental data, confirming that the observed signal reduction originates from a destructive interference at specific BP thicknesses. The G‐BP heterostructure formation enabled successful laser‐triggered functional group patterning of graphene under mild conditions (1 mW, 3 s) within the defined BP thickness and laser irradiation regime, evidenced by a prominent emergence of the defect‐induced *D* band of graphene (*I_D_*/*I_G_* ≈ 0.8) and consistent with previously observed covalent added binding to graphene. The functionalization efficiency was lower than on the Si/SiO_2_ substrate (*I_D_*/*I_G_* ≈ 1.2), suggesting a limited effectiveness for an antaratopic backbonding of graphene towards the underlying BP. T‐dependent Raman experiments on functionalized G‐BP heterostructures revealed an irreversible behavior upon heating from room temperature to 400°C. While the BP signals remained stable below 150°C, they progressively decreased and disappeared completely at 200°C, accompanied by an increased background fluorescence and the broadening of all graphene modes. These findings demonstrate that the graphene sheet acts as a physical barrier, trapping reactive BP decomposition fragments that subsequently react with the graphene in an uncontrolled manner. This trapped‐fragment mechanism establishes additional defects in the graphene lattice, resulting in the observed spectral changes exclusively in regions where BP was previously located. Overall, this work establishes G‐BP heterostructures as a viable platform for controlled laser‐induced functionalization while highlighting substrate‐dependent efficiency limitations and the protective, yet constraining, role of the superjacent graphene layer.

## 4 Experimental

### 4.1 Sample Preparation

BP crystals were mechanically exfoliated using a sticky polymer tape and transferred to Si/SiO_2_ to yield few‐layer BP. CVD‐grown graphene was transferred onto these wafers under heat by a dry transfer technique to yield G‐BP heterostructures, and the protective PMMA layer on top of the graphene was removed using acetone vapor.

### 4.2 Solvent Preparation

Commercially available anhydrous THF was purified beforehand. The solvent was pump‐freezed at least five times to exclude the presence of oxygen.

### 4.3 Scanning Probe Microscopy

AFM images were recorded using a Bruker Dimension Icon 3 microscope in ScanAsyst Air mode. AFM topography images were obtained with Bruker ScanAsyst‐Air silicon tips on nitride levers coated with reflective aluminum and a spring constant of 0.4 N/m, at a resolution of either 512 × 512 or 1024 × 1024 pixels and with a scan rate of 0.4 Hz. Bruker SCM‐PIT‐V2 probes with platinum‐iridium coating on antimony‐doped Si cantilever were used to obtain KPFM images resolved by either 512 × 512 pixels or 1024 × 1024 pixels and scan rates of either 0.2 or 0.3 Hz were used.

### 4.4 Raman Spectroscopy

Raman spectra and maps were acquired on a WITec alpha300 R confocal microscope equipped with an automated XYZ stage. All Raman measurements were conducted using an excitation wavelength of 532 nm, if not stated otherwise. The parameters were selected according to the individual experiment. The read‐out Raman mappings were recorded with 3 mW and with integrations times of 0.5 s or 1 s and T‐dependent Raman measurements were performed in a Linkam stage chamber mounted to the Raman microscope stage.

### 4.5 Scanning Electron Microscopy

SEM images and EDS measurements were performed on a FEI‐Helios NanoLab 600i FIB focused ion beam/scanning electron microscope (FIB/SEM). An Oxford Instruments X‐Max detector integrated in the system was used for EDS experiments.

### 4.6 Methods

AI engines were used for linguistic and stylistic corrections of the manuscript.

## Conflicts of Interest

The authors declare no conflicts of interest.

## Supporting information



The authors have cited additional references within the Supporting Information [71, 72, 73, 74, 75].
**Supporting File**: chem71127‐sup‐0001‐SuppMat.pdf.

## Data Availability

The data that support the findings of this study are available at Zenodo at https://doi.org/10.5281/zenodo.17854837.

## References

[1] K. S. Novoselov , A. K. Geim , S. V. Morozov , et al., “Electric Field Effect in Atomically Thin Carbon Films,” Science 306, no. 5696 (2004): 666–669, 10.1126/science.1102896. 15499015

[2] N. R. Glavin , R. Rao , V. Varshney , et al., “Emerging Applications of Elemental 2D Materials,” Advanced Materials 32, no. 7 (2020): 1904302, 10.1002/adma.201904302. 31667920

[3] X. Zou , Y. Xu , and W. Duan , “2D Materials: Rising Star for Future Applications,” Innovation 2, no. 2 (2021): 100115. 34557763 10.1016/j.xinn.2021.100115 PMC8454666

[4] F. Xia , H. Wang , and Y. Jia , “Rediscovering Black Phosphorus as an Anisotropic Layered Material for Optoelectronics and Electronics,” Nature Communications 5, no. 1 (2014): 4458, 10.1038/ncomms5458. 25041752

[5] S. Wu , K. S. K. N. Hui , and K. S. K. N. Hui , “2D Black Phosphorus: From Preparation to Applications for Electrochemical Energy Storage,” Advanced Science 5, no. 5 (2018): 1700491, 10.1002/advs.201700491. 29876201 PMC5980130

[6] M. Buscema , D. J. Groenendijk , S. I. Blanter , G. A. Steele , H. S. J. J. van der Zant , and A. Castellanos‐Gomez , “Fast and Broadband Photoresponse of Few‐Layer Black Phosphorus Field‐Effect Transistors,” Nano Letters 14, no. 6 (2014): 3347–3352, 10.1021/nl5008085. 24821381

[7] J. D. Wood , S. A. Wells , D. Jariwala , et al., “Effective Passivation of Exfoliated Black Phosphorus Transistors Against Ambient Degradation,” Nano Letters 14, no. 12 (2014): 6964–6970, 10.1021/nl5032293. 25380142

[8] M. Buscema , D. J. Groenendijk , G. A. Steele , H. S. J. van der Zant , and A. Castellanos‐Gomez , “Photovoltaic Effect in Few‐Layer Black Phosphorus PN Junctions Defined by Local Electrostatic Gating,” Nature Communications 5, no. 1 (2014): 4651, 10.1038/ncomms5651. 25164986

[9] S. Lin , S. Liu , Z. Yang , et al., “Solution‐Processable Ultrathin Black Phosphorus as an Effective Electron Transport Layer in Organic Photovoltaics,” Advanced Functional Materials 26, no. 6 (2016): 864–871, 10.1002/adfm.201503273.

[10] M. Batmunkh , M. Bat‐Erdene , and J. G. Shapter , “Black Phosphorus: Synthesis and Application for Solar Cells,” Advanced Energy Materials 8, no. 5 (2018): 1701832, 10.1002/aenm.201701832.

[11] X. Gong , L. Guan , Q. Li , et al., “Black Phosphorus Quantum Dots in Inorganic Perovskite Thin Films for Efficient Photovoltaic Application,” Science Advances 6, no. 15 (2020): eaay5661, 10.1126/sciadv.aay5661. 32300650 PMC7148097

[12] R. Fei , A. Faghaninia , R. Soklaski , J. A. Yan , C. Lo , and L. Yang , “Enhanced Thermoelectric Efficiency via Orthogonal Electrical and Thermal Conductances in Phosphorene,” Nano Letters 14, no. 11 (2014): 6393–6399, 10.1021/nl502865s. 25254626

[13] J. Zhang , H. J. Liu , L. Cheng , et al., “Phosphorene Nanoribbon as a Promising Candidate for Thermoelectric Applications,” Scientific Reports 4, no. 1 (2014): 1–8. 10.1038/srep06452 PMC4171703 25245326

[14] P. Li , D. Zhang , J. Liu , H. Chang , Y. Sun , and N. Yin , “Air‐Stable Black Phosphorus Devices for Ion Sensing,” ACS Applied Materials & Interfaces 7, no. 44 (2015): 24396–24402, 10.1021/acsami.5b07712. 26501864

[15] A. N. Abbas , B. Liu , L. Chen , et al., “Black Phosphorus Gas Sensors,” ACS Nano 9, no. 5 (2015): 5618–5624, 10.1021/acsnano.5b01961. 25945545

[16] M. Engel , M. Steiner , and P. Avouris , “Black Phosphorus Photodetector for Multispectral, High‐Resolution Imaging,” Nano Letters 14, no. 11 (2014): 6414–6417, 10.1021/nl502928y. 25299161

[17] N. Youngblood , C. Chen , S. J. Koester , and M. Li , “Waveguide‐Integrated Black Phosphorus Photodetector With High Responsivity and Low Dark Current,” Nature Photonics 9, no. 4 (2015): 247–252, 10.1038/nphoton.2015.23.

[18] Y. Chen , G. Jiang , S. Chen , et al., “Mechanically Exfoliated Black Phosphorus as a New Saturable Absorber for both Q‐Switching and Mode‐Locking Laser Operation,” Optics Express 23, no. 10 (2015): 12823, 10.1364/OE.23.012823. 26074536

[19] H. Uk Lee , S. C. Lee , J. Won , et al., “Stable Semiconductor Black Phosphorus (BP)@Titanium Dioxide (TiO_2_) Hybrid Photocatalysts,” Scientific Reports 5, no. 1 (2015): 8691, 10.1038/srep08691. 25732720 PMC4346807

[20] M. Z. Rahman , C. W. Kwong , K. Davey , and S. Z. Qiao , “2D Phosphorene as a Water Splitting Photocatalyst: Fundamentals to Applications,” Energy & Environmental Science 9, no. 3 (2016): 709–728, 10.1039/C5EE03732H.

[21] R. He , J. Hua , A. Zhang , et al., “Molybdenum Disulfide–Black Phosphorus Hybrid Nanosheets as a Superior Catalyst for Electrochemical Hydrogen Evolution,” Nano Letters 17, no. 7 (2017): 4311–4316, 10.1021/acs.nanolett.7b01334. 28605201

[22] J. O. Island , G. A. Steele , H. S. J. van der Zant , A. Castellanos‐Gomez , H. S. J. Van Der Zant , and A. Castellanos‐Gomez , “Environmental Instability of Few‐Layer Black Phosphorus,” 2D Materials 2 (2015): 011002.

[23] G. Abellán , S. Wild , V. Lloret , et al., “Fundamental Insights Into the Degradation and Stabilization of Thin Layer Black Phosphorus,” Journal of the American Chemical Society 139, no. 30 (2017): 10432–10440, 10.1021/jacs.7b04971. 28675300 PMC5578363

[24] T. Zhang , Y. Wan , H. Xie , et al., “Degradation Chemistry and Stabilization of Exfoliated Few‐Layer Black Phosphorus in Water,” Journal of the American Chemical Society 140, no. 24 (2018): 7561–7567, 10.1021/jacs.8b02156. 29575904

[25] S. Gamage , A. Fali , N. Aghamiri , L. Yang , P. D. Ye , and Y. Abate , “Reliable Passivation of Black Phosphorus by Thin Hybrid Coating,” Nanotechnology 28, no. 26 (2017): 265201, 10.1088/1361-6528/aa7532. 28548048

[26] Y. Y. Illarionov , M. Waltl , G. Rzepa , et al., “Long‐Term Stability and Reliability of Black Phosphorus Field‐Effect Transistors,” ACS Nano 10, no. 10 (2016): 9543–9549, 10.1021/acsnano.6b04814. 27704779

[27] J.‐S. Kim , Y. Liu , W. Zhu , et al., “Toward Air‐Stable Multilayer Phosphorene Thin‐Films and Transistors,” Scientific Reports 5, no. 1 (2015): 8989, 10.1038/srep08989. 25758437 PMC4355728

[28] J. Kim , S. K. Baek , K. S. Kim , Y. J. Chang , and E. J. Choi , “Long‐Term Stability Study of Graphene‐Passivated Black Phosphorus Under Air Exposure,” Current Applied Physics 16, no. 2 (2016): 165–169, 10.1016/j.cap.2015.11.010.

[29] R. A. Doganov , E. C. T. O'Farrell , S. P. Koenig , et al., “Transport Properties of Pristine Few‐Layer Black Phosphorus by van Der Waals Passivation in an Inert Atmosphere,” Nature Communications 6, no. 1 (2015): 6647, 10.1038/ncomms7647. 25858614

[30] J. Yoon and Z. Lee , “Effective Passivation of Black Phosphorus Under Ambient Conditions,” Applied Microscopy 47, no. 3 (2017): 176–186, 10.9729/AM.2017.47.3.176.

[31] L. Zhang , L.‐F. Gao , L. Li , et al., “Negatively Charged 2D Black Phosphorus for Highly Efficient Covalent Functionalization,” Materials Chemistry Frontiers 2, no. 9 (2018): 1700–1706, 10.1039/C8QM00237A.

[32] Z. Sofer , J. Luxa , D. Bouša , et al., “The Covalent Functionalization of Layered Black Phosphorus by Nucleophilic Reagents,” Angewandte Chemie International Edition 56, no. 33 (2017): 9891–9896, 10.1002/anie.201705722. 28631314

[33] S. Wild , M. Fickert , A. Mitrovic , et al., “Lattice Opening Upon Bulk Reductive Covalent Functionalization of Black Phosphorus,” Angewandte Chemie International Edition 58, no. 17 (2019): 5763–5768, 10.1002/anie.201811181. 30675972 PMC7318246

[34] X. Liu , Y. Bai , J. Xu , et al., “Robust Amphiphobic Few‐Layer Black Phosphorus Nanosheet With Improved Stability,” Advanced Science 6, no. 23 (2019): 1901991, 10.1002/advs.201901991. 31832324 PMC6891918

[35] M. van Druenen , F. Davitt , T. Collins , et al., “Covalent Functionalization of Few‐Layer Black Phosphorus Using Iodonium Salts and Comparison to Diazonium Modified Black Phosphorus,” Chemistry of Materials 30, no. 14 (2018): 4667–4674, 10.1021/acs.chemmater.8b01306.

[36] Y. Liu , P. Gao , T. Zhang , et al., “Azide Passivation of Black Phosphorus Nanosheets: Covalent Functionalization Affords Ambient Stability Enhancement,” Angewandte Chemie 131, no. 5 (2019): 1493–1497, 10.1002/ange.201813218. 30536864

[37] Y. Zhao , H. Wang , H. Huang , et al., “Surface Coordination of Black Phosphorus for Robust Air and Water Stability,” Angewandte Chemie 128, no. 16 (2016): 5087–5091, 10.1002/ange.201512038. 26968443

[38] L. Wu , J. Wang , J. Lu , et al., “Lanthanide‐Coordinated Black Phosphorus,” Small 14, no. 29 (2018): 1801405, 10.1002/smll.201801405. 29931730

[39] H. Hu , H. Gao , L. Gao , et al., “Covalent Functionalization of Black Phosphorus Nanoflakes by Carbon Free Radicals for Durable Air and Water Stability,” Nanoscale 10, no. 13 (2018): 5834–5839, 10.1039/C7NR06085H. 29542740

[40] C. R. Ryder , J. D. Wood , S. A. Wells , et al., “Covalent Functionalization and Passivation of Exfoliated Black Phosphorus via Aryl Diazonium Chemistry,” Nature Chemistry 8, no. 6 (2016): 597–602, 10.1038/nchem.2505. 27219705

[41] V. Strong , S. Dubin , M. F. El‐Kady , et al., “Patterning and Electronic Tuning of Laser Scribed Graphene for Flexible all‐Carbon Devices,” ACS Nano 6, no. 2 (2012): 1395–1403, 10.1021/nn204200w. 22242925

[42] R. Ye , D. K. James , J. M. Tour , R. Ye , D. K. James , and M. Tour , “Laser‐Induced Graphene: From Discovery to Translation,” Advanced Materials 31, no. 1 (2019): 1803621, 10.1002/adma.201803621. 30368919

[43] K. F. Edelthalhammer , D. Dasler , L. Jurkiewicz , et al., “Covalent 2D‐Engineering of Graphene by Spatially Resolved Laser Writing/Reading/Erasing,” Angewandte Chemie International Edition 59, no. 51 (2020): 23329–23334, 10.1002/anie.202006874. 32808699 PMC7756404

[44] T. Wei , M. Kohring , H. B. Weber , F. Hauke , and A. Hirsch , “Molecular Embroidering of Graphene,” Nature Communications 12, no. 1 (2021): 552, 10.1038/s41467-020-20651-w. PMC7822905 33483478

[45] T. Nagel , S. Wolff , S. Feng , et al., “Towards Precision Controlled 2D Functional Group Patterning of Graphene via Laser Writing,” Carbon 241 (2025): 120376, 10.1016/j.carbon.2025.120376.

[46] K. P. Loh , Q. Bao , P. K. Ang , and J. Yang , “The Chemistry of Graphene,” Journal of Materials Chemistry 20, no. 12 (2010): 2277, 10.1039/b920539j.

[47] X. Chen , M. Assebban , M. Kohring , et al., “Laser‐Triggered Bottom‐Up Transcription of Chemical Information: Toward Patterned Graphene/MoS_2_ Heterostructures,” Journal of the American Chemical Society 144, no. 22 (2022): 9645–9650, 10.1021/jacs.2c00642. 35617156 PMC9185739

[48] J. Lu , J. Wu , A. Carvalho , et al., “Bandgap Engineering of Phosphorene by Laser Oxidation Toward Functional 2D Materials,” ACS Nano 9, no. 10 (2015): 10411–10421, 10.1021/acsnano.5b04623. 26364647

[49] X. Liu , K. Chen , S. A. Wells , et al., “Scanning Probe Nanopatterning and Layer‐by‐Layer Thinning of Black Phosphorus,” Advanced Materials 29, no. 1 (2017): 1604121, 10.1002/adma.201604121. 27813243

[50] L. Li , Y. Yu , G. J. Ye , et al., “Black Phosphorus Field‐Effect Transistors,” Nature Nanotechnology 9, no. 5 (2014): 372–377, 10.1038/nnano.2014.35. 24584274

[51] J. Plutnar , Z. Sofer , and M. Pumera , “Products of Degradation of Black Phosphorus in Protic Solvents,” ACS Nano 12, no. 8 (2018): 8390–8396, 10.1021/acsnano.8b03740. 30106272

[52] Y. Wang , B. Yang , B. Wan , et al., “Degradation of Black Phosphorus: A Real‐Time 31P NMR Study,” 2D Materials 3, no. 3 (2016): 035025.

[53] X. Ling , H. Wang , S. Huang , F. Xia , and M. S. Dresselhaus , “The Renaissance of Black Phosphorus,” Proceedings of the National Academy of Sciences 112, no. 15 (2015): 4523–4530, 10.1073/pnas.1416581112. PMC4403146 25820173

[54] T. Ahmed , S. Balendhran , M. N. Karim , et al., “Degradation of Black Phosphorus Is Contingent on UV–Blue Light Exposure,” Npj 2D Materials and Applications 1, no. 1 (2017): 18.

[55] R. A. Schäfer , K. Weber , M. Koleśnik‐Gray , et al., “Substrate‐Modulated Reductive Graphene Functionalization,” Angewandte Chemie International Edition 55, no. 47 (2016): 14858–14862, 10.1002/anie.201607427. 27781343

[56] K. Amsharov , D. I. Sharapa , O. A. Vasilyev , et al., “Fractal‐Seaweeds Type Functionalization of Graphene,” Carbon 158 (2020): 435–448, 10.1016/j.carbon.2019.11.008.

[57] J. Kang , D. Jariwala , C. R. Ryder , et al., “Probing Out‐of‐Plane Charge Transport in Black Phosphorus With Graphene‐Contacted Vertical Field‐Effect Transistors,” Nano Letters 16, no. 4 (2016): 2580–2585, 10.1021/acs.nanolett.6b00144. 26950174

[58] M. Li , N. Muralidharan , K. Moyer , and C. L. Pint , “Solvent Mediated Hybrid 2D Materials: Black Phosphorus—Graphene Heterostructured Building Blocks Assembled for Sodium Ion Batteries,” Nanoscale 10, no. 22 (2018): 10443–10449, 10.1039/C8NR01400K. 29796515

[59] S. Akhavan , A. Ruocco , G. Soavi , et al., “Graphene‐Black Phosphorus Printed Photodetectors,” 2D Materials 10, no. 3 (2023): 035015.

[60] Y. Liu , I. Yudhistira , M. Yang , et al., “Phonon‐Mediated Colossal Magnetoresistance in Graphene/Black Phosphorus Heterostructures,” Nano Letters 18, no. 6 (2018): 3377–3383, 10.1021/acs.nanolett.8b00155. 29726254

[61] T. Nagel , F. Hauke , and A. Hirsch , “Sequential Laser “Writing” for Mulit‐Component Functionalization of Graphene,”.

[62] A. Favron , E. Gaufrès , F. Fossard , et al., “Photooxidation and Quantum Confinement Effects in Exfoliated Black Phosphorus,” Nature Materials 14, no. 8 (2015): 826–832, 10.1038/nmat4299. 26006004

[63] L. G. Cançado , A. Jorio , E. H. M. M. Ferreira , et al., “Quantifying Defects in Graphene via Raman Spectroscopy at Different Excitation Energies,” Nano Letters 11, no. 8 (2011): 3190–3196. 21696186 10.1021/nl201432g

[64] M. M. Lucchese , F. Stavale , E. H. M. Ferreira , et al., “Quantifying Ion‐Induced Defects and Raman Relaxation Length in Graphene,” Carbon 48, no. 5 (2010): 1592–1597, 10.1016/j.carbon.2009.12.057.

[65] Q. H. Wang , Z. Jin , K. K. Kim , et al., “Understanding and Controlling the Substrate Effect on Graphene Electron‐Transfer Chemistry via Reactivity Imprint Lithography,” Nature Chemistry 4, no. 9 (2012): 724–732, 10.1038/nchem.1421. 22914193

[66] K. C. Knirsch , R. A. Schäfer , F. Hauke , and A. Hirsch , “Mono‐ and Ditopic Bisfunctionalization of Graphene,” Angewandte Chemie International Edition 55, no. 19 (2016): 5861–5864, 10.1002/anie.201511807. 27037789

[67] T. Dierke , D. Dasler , T. Nagel , F. Hauke , A. Hirsch , and J. Maultzsch , “Spatial Control of Graphene Functionalization by Patterning a 2D Substrate: Implications for Graphene Based van‐Der‐Waals Heterostructures,” ACS Applied Nano Materials 5, no. 4 (2022): 4966–4971, 10.1021/acsanm.1c04559.

[68] P. Vecera , K. Edelthalhammer , F. Hauke , and A. Hirsch , “Reductive Arylation of Graphene: Insights Into a Reversible Carbon Allotrope Functionalization Reaction,” Physica Status Solidi (b) 251, no. 12 (2014): 2536–2540, 10.1002/pssb.201451315.

[69] T. Wei , M. Kohring , M. Chen , et al., “Highly Efficient and Reversible Covalent Patterning of Graphene: 2D‐Management of Chemical Information,” Angewandte Chemie International Edition 59, no. 14 (2020): 5602–5606, 10.1002/anie.201914088. 31833618 PMC7154694

[70] J. Kang , J. D. Wood , S. A. Wells , et al., “Solvent Exfoliation of Electronic‐Grade, Two‐Dimensional Black Phosphorus,” ACS Nano 9, no. 4 (2015): 3596–3604, 10.1021/acsnano.5b01143. 25785299

[71] T. Dierke , Spatially Controlled Functionalization of Graphene and Twisted Bilayer Graphene (Friedrich‐Alexander‐University, 2025).

[72] H. Anders , Duenne Schichten Fuer Die Optik (Wissenschaftliche Verlagsgesellschaft Stuttgart, 1965).

[73] P. Blake , E. W. Hill , A. H. Castro Neto , et al., “Making Graphene Visible,” Applied Physics Letters 91, no. 6 (2007): 063124.

[74] J. Henrie , S. Kellis , S. M. Schultz , and A. Hawkins , “Electronic Color Charts for Dielectric Films on Silicon,” Optics Express 12, no. 7 (2004): 1464, 10.1364/OPEX.12.001464. 19474970

[75] J. Kim , J.‐U. Lee , J. Lee , et al., “Anomalous Polarization Dependence of Raman Scattering and Crystallographic Orientation of Black Phosphorus,” Nanoscale 7, no. 44 (2015): 18708–18715, 10.1039/C5NR04349B. 26503032

