# Supplementary material for: Laser‐Induced Chemical Patterning of Graphene‐Black Phosphorus Hybrids
Source: Chemistry. 2026 May 14;32(22):e71127. doi: 10.1002/chem.71127 (PMC13250376; doi:10.1002/chem.71127)
Supplement: Supplementary file 1 — The authors have cited additional references within the Supporting Information [71, 72, 73, 74, 75]. Supporting File: chem71127‐sup‐0001‐SuppMat.pdf. [file CHEM-32-e71127-s001.pdf]

Supporting Information

Laser-Induced Chemical Patterning of Graphene-Black Phosphorus Hybrids

Jasmin Eisenkolb,[a] Mhamed Assebban,[a] Tobias Dierke,[b] Janina Maultzsch,[b] Andreas Hirsch*[a] and Frank Hauke*[a]

[a] Department of Chemistry and Pharmacy & Center of Advanced Materials and Processes (ZMP), Friedrich-Alexander-Universität Erlangen-Nürnberg, Nikolaus-Fiebiger-Str. 10, 91058 Erlangen, Germany

[b] Chair of Experimental Physics, Friedrich-Alexander-Universität Erlangen-Nürnberg, Staudtstraße 7, 91058 Erlangen, Germany

E-mail: frank.hauke@fau.de; andreas.hirsch@fau.de

Table of Contents

Instrumental 3

Materials 4

Experimental 4

Supplementary Figures 7

S1: Laser irradiation of a BP flake using varying laser powers 7

S2: Energy-dispersive X-ray mapping of a laser-irradiated BP flake 8

S3: Reference sample using low laser power irradiation below 15 mW 9

S4: Reference experiment on around 10 nm thin BP flakes towards their tendency for photo-induced oxidation 10

S5: Irradiation of a BP nanosheet using a 633 nm laser 11

S6: Energy-dispersive X-ray mapping of a BP flake after the laser-triggered covalent functionalization using Cl-DBPO 12

S7: Mean Raman spectra of the pristine G-BP heterostructure under mild laser irradiation conditions 13

S8: Single Raman spectrum of the pristine G-BP heterostructure 14

Theoretical calculations 15

References 16

Instrumental

Scanning probe microscopy was carried out using a Bruker Dimension Icon 3 microscope in ScanAsyst Air or AM-KPFM (Amplitude-Modulated Kelvin Probe Force Microscopy) mode. AFM topography images were obtained with Bruker ScanAsyst-Air silicon tips on nitride levers coated with reflective aluminum and a spring constant of 0.4 N/m, at a resolution of either 512×512 or 1024×1024 pixels and with a scan rate of 0.4 Hz. Bruker SCM-PIT-V2 probes with platinum-iridium coating on antimony-doped Si cantilevers were used to obtain KPFM images resolved by either 512x512 pixels or 1024x1024 pixels. Scan rates of 0.2 or 0.3 Hz were used.

Raman measurements were acquired on a WITec alpha300 R confocal microscope equipped with an automated XYZ stage. The optical beam path of the Raman system was coupled with an optical microscope, which in combination with the built-in Easy Link function, allows for a straightforward and facile positioning on samples and the monitoring of conducted direct laser writings. All Raman measurements were conducted using a 100x Zeiss EC "Epiplan-Neofluar" (N_A = 0.90) or a long-distance 50x Zeiss EC "Epiplan-Neofluar" DIC (N_A = 0.55) where indicated, and a DPSS laser with an excitation wavelength of λ = 532 nm. Whenever wavelength-dependent studies were presented, two additional lasers with λ = 457 nm and λ = 633 nm were used. In all cases presented in this manuscript, a grating of 600 grooves/mm was used and laser power and irradiation times were changed depending on the experiment as indicated in the respective section. Data were evaluated utilizing the WITec Project Software.

By coupling the WITec alpha300R Raman instrument to a THMS600 Linkam Heating and Freezing stage, temperature-dependent measurements were performed. The Linkam stage allows measurements in a temperature range between -196 °C and 600 °C and is mounted onto the XYZ stage of the Raman instrument, enabling the samples inside the Linkam stage chamber to be heated and cooled down directly under the optical microscope, which allows direct monitoring of the sample during the heating (or cooling) process. Raman measurements are made possible due to the top-window of the Linkam stage that allows light to pass through. Heating rates were controlled using the Linkam software.

Scanning electron microscopy (SEM) and **energy dispersive X-ray spectroscopy (EDS)** were performed using a FEI-Helios NanoLab 600i FIB focused ion beam/scanning electron microscope (FIB/SEM). An Oxford Instruments X-Max detector integrated in the system was used for EDS experiments.

Materials

Bulk black phosphorus crystals were purchased from Smart elements (99.998% purity). All chemicals used were purchased from either Sigma Aldrich or Carl Roth and anhydrous THF was pump-frozen at least five times to exclude oxygen when used as a solvent inside the glovebox. CVD-grown monolayer graphene was purchased from ACS Materials Inc. as trivial transfer graphene with PMMA coating. Silicon wafers with a 300 nm thick silicon oxide top-layer and silicon carbide wafers were obtained from Fraunhofer e.V. and were cut into 0.5 x 0.5 cm substrates. The two presented PDIs were synthesized by the Hirsch and Sastre groups.

Experimental

Cleaning of Si/SiO_2 and SiC wafers

Si/SiO_2 wafers with a 300 nm thick oxide layer or SiC wafers were cleaned by sonication in isopropyl alcohol (IPA) for 1 min and subsequent washing with acetone and IPA using a spin-coater.

Mechanical exfoliation of BP

For all experiments, mechanically exfoliated BP was used. Commercially available bulk BP crystals were deposited on a sticky polymer tape and by applying a simple "press and peel"-technique, few-layer BP was obtained. The as-obtained BP flakes were transferred onto pre-cleaned Si/SiO_2 (or SiC) substrates. Depending on the cleanliness of the surface after the transfer, a second sonication step in IPA was performed.

Laser irradiation of mechanically exfoliated BP

To investigate the effect of laser irradiation on pristine BP flakes, mechanically exfoliated BP nanosheets were transferred on pre-cleaned SiC. This substrate was selected as it allows for a facilitated investigation of the oxygen content on the surface after irradiation by EDS measurements without the interference of intrinsic oxygen in the substrate. Analogous to the direct laser writing procedure, two optically selected flakes were locally irradiated with a green laser (λ = 532 nm) with varying laser powers, while the irradiation time was kept constant at 3 s. Area mappings in the shape of a line were performed for each set of parameters (3 s irradiation time, 0.5 µm step size between points, 2 µm width of the "line", length was selected depending on the flake) and the laser power was

increased step-wise from 1 mW up to 22 mW, which corresponds to the maximum laser output of our Raman system (see **Figure S1**). AFM images were recorded before and after the irradiation for comparison. The same set of experiments was repeated on thinner flakes on another sample to gain insights into the thickness dependency of the process. In accordance with the investigation of irradiating pristine BP nanosheets with varying laser powers, the wavelength dependency was likewise analyzed. Therefore, mechanically exfoliated BP was transferred on pre-cleaned Si/SiO_2 wafers and a suitable flake was selected by optical contrast. Similar to before, the flake was irradiated locally with a laser, however, in this case all parameters were kept constant (15 mW laser power, 3 s irradiation time, 0.5 µm step size between points, 2 µm width of the "line", length was selected depending on the flake) and the wavelength of the laser was varied. Overall, three lasers, blue (λ = 457 nm), green (λ = 532 nm) and red (λ = 633 nm), were tested and investigated with regard to their ability to cause photo-oxidation of the selected BP. AFM images were recorded before and after irradiation.

Covalent functionalization of BP

Initially, wafers with mechanically exfoliated BP on Si/SiO_2 were spin-coated or drop-casted with a certain compound solution to create a thin film of a highly reactive substance, *bis*(4-chlorobenzoyl)peroxide (Cl-DBPO). To achieve covalent functionalization, the optimized laser writing procedure that has been already established for the attachment of organic moieties to monolayer graphene was adapted.[1] The laser writing process was carried out using a green laser (λ = 532 nm), where specific areas of the coated wafer with visible BP flakes were irradiated with varying parameters. Different approaches for the formation of a homogenous and sufficiently concentrated coating were tested by varying the coating method or changing the concentration of the Cl-DBPO solution and the corresponding solvents. Three approaches have been tested. First, analogous to [1], 2.5 mg of Cl-DBPO were dissolved in 0.5 mL Et_2O (c = 1.6×10^{-2} mol/L) and spin-coated on the prepared BP sample to create the coating, which was, in comparison to graphene, rather inhomogeneous and thick according to optical characterization. Therefore, in a second attempt, only 1.5 mg of the reagent was dissolved in 0.5 mL of THF (c = 9.6×10^{-3} mol/L) and it was drop-casted on the BP sample. Using THF offered the possibility to transfer the whole process into an Ar-filled glovebox, allowing the BP flakes to remain under inert conditions before coating them. The obtained coating is still rather thick and, depending on the spot, partially inhomogeneous. In a final attempt, a PMMA-assisted spin-coating technique was used to afford a more homogeneous and thin coating by spin-coating Cl-DBPO in THF mixed with PMMA (c = 10^{-3} mol/L) onto Si/SiO_2 wafers with mechanically exfoliated BP. After the laser irradiation, the samples were thoroughly washed by immersing the wafers in clean solvent (Et_2O in case of the first coating prepared in Et_2O and THF for all coating

prepared by dissolving Cl-DBPO in THF) for either 30 min or overnight and after subsequent rinsing and air-drying, the irradiated areas of the wafers were analyzed by Raman spectroscopy and AFM.

Dry transfer of CVD graphene and formation of the G-BP heterostructure

Trivial Transfer Graphene with a protective PMMA layer was transferred from its carrier material to a transparent polymer film and cut into smaller pieces. These pieces were introduced into the glovebox and were pressed under constant heating at 125 °C onto previously prepared and pre-heated Si/SiO_2 wafers with mechanically exfoliated BP. After 40 min, the small polymer pieces were slowly and carefully pulled up and thereby removed, while the monolayer graphene (still with PMMA) was transferred to the Si/SiO_2 wafers with mechanically exfoliated BP. Subsequently, the PMMA layer was removed by exposing the wafers to acetone vapor for 2 hours.

Laser irradiation of the G-BP heterostructure

As described above, CVD-grown graphene was transferred onto Si/SiO_2 wafers with mechanically exfoliated BP nanosheets under heat. The PMMA was removed by immersing the wafer in THF. A suitable BP flake covered with graphene was selected by optical contrast. In our Raman setup, the same G-BP heterostructure was selected and rectangular patterns were irradiated with the green laser (λ = 532 nm). The laser power was varied between 1 mW and 22 mW to investigate the laser-induced photo-oxidation of G-BP hybrids, using a constant irradiation time of 3 s and a step size of 0.5 µm for all patterns. The size of the rectangle was adjusted according to the lateral dimensions of the BP nanosheet. AFM images were recorded before and after irradiation.

Covalent functionalization of the G-BP heterostructure

Similar to the already mentioned covalent functionalization of BP, the covalent functionalization of the G-BP heterostructure was achieved by applying the laser writing procedure. Therefore, CVD graphene was transferred onto Si/SiO_2 wafers with mechanically exfoliated BP as described above and a thin film of Cl-DBPO (c = 10^{-3} mol/L in THF) was drop-casted onto the samples. The laser writing was performed using a 532 nm laser with varying laser irradiation parameters as indicated in each case. Subsequently, the residual coating was washed away by immersing the wafers in IPA and sonicating them cautiously. The irradiated areas were analyzed by Raman spectroscopy and AFM. In addition, temperature-dependent defunctionalization of the covalently grafted addends was attempted by stepwise heating of the sample up to 400 °C, while simultaneously monitoring the changes by Raman spectroscopy.

Supplementary Figures

S1: Laser irradiation of a BP flake using varying laser powers

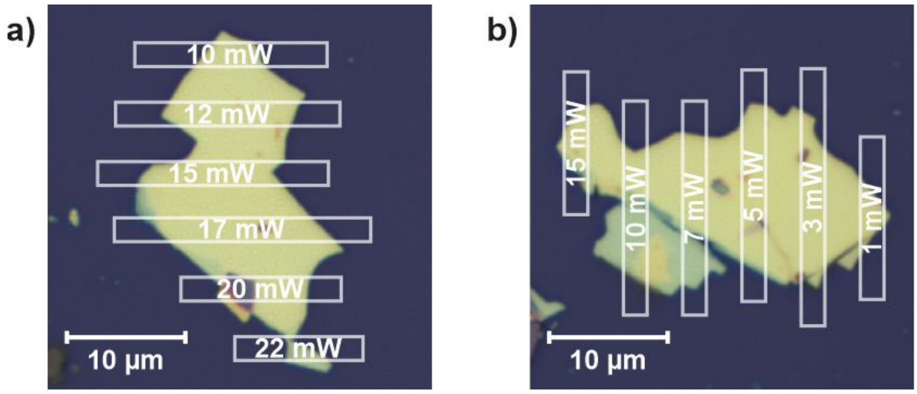

Figure S1*. Graphical description of the experimental procedure of irradiating a 32 nm thick BP flake with a 532 nm laser using varying laser powers. The irradiated areas are indicated by white rectangles with their corresponding laser powers.*

Overall, two flakes with the same height were selected on the same sample to enable more possibilities for parameter variation. The areas that were irradiated with a green laser, are marked with a white rectangle. Between each illuminated line, a distance of 5 µm was kept to guarantee no overlap between the lines irradiated with varying laser powers and for a non-irradiated BP reference later on. All writing parameters were kept constant for all lines (532 nm laser wavelength, 3 s irradiation time, 0.5 µm step size between irradiated points, 2 µm width), except for the exact length of the lines due to the distinct shape of the irradiated BP flake and the corresponding laser power, indicated in the respective white rectangle. The optical image in (a) shows the flake irradiated in the high laser power regime (> 10 mW), that is shown in **Figure 1** in the manuscript. Similarly, the second flake in the optical image (b) was irradiated with low laser powers (< 10 mW) as indicated.

S2: Energy-dispersive X-ray mapping of a laser-irradiated BP flake

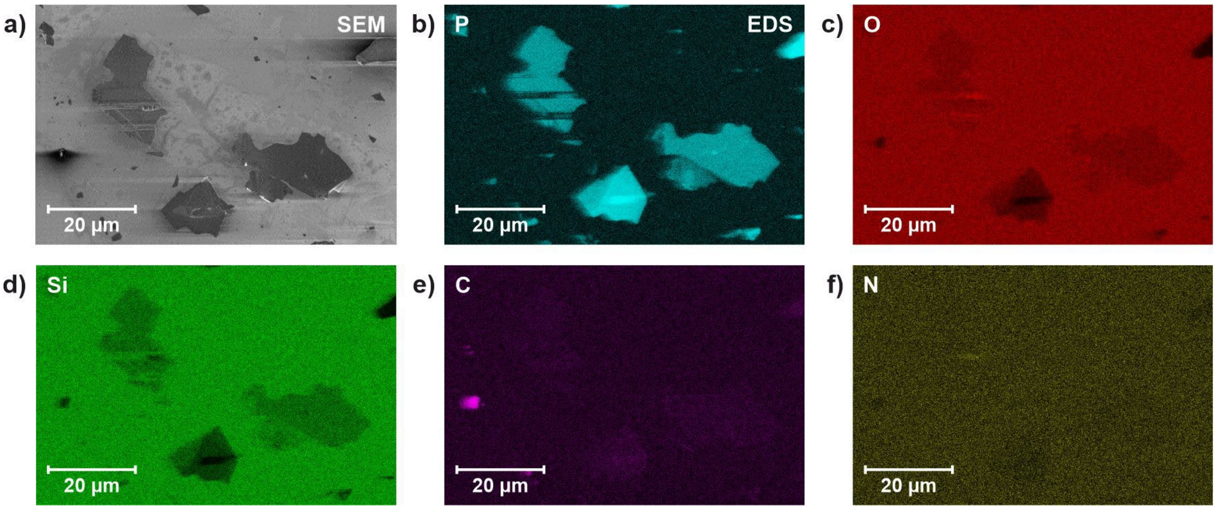

***Figure S2**. Recorded scanning electron (SEM) image **(a)** of the two irradiated BP flakes shown in **Figure S1**. Corresponding energy-dispersive X-ray (EDX) mappings depicting the distribution of pre-selected elements, in this case of phosphorus **(b)**, oxygen **(c)**, silicon **(d)**, carbon **(e)** and nitrogen **(f)**.*

S3: Reference sample using low laser power irradiation below 15 mW

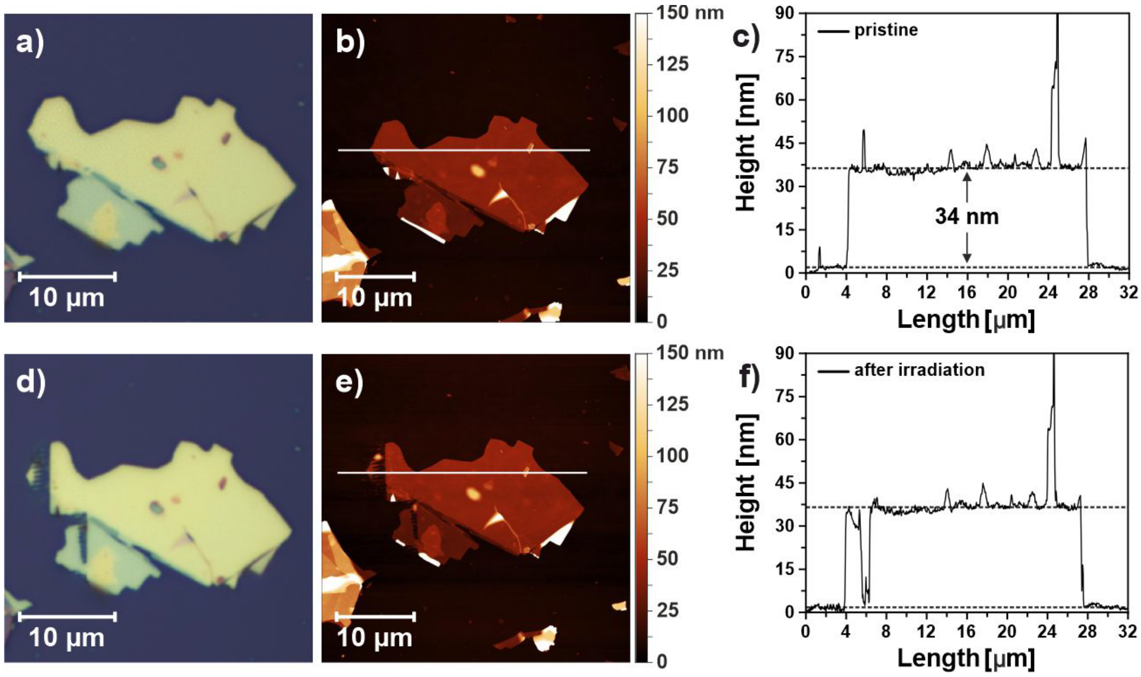

Figure S3. *Characterization and comparison of a laser irradiated BP flake to its pristine counterpart. Optical* ***(a)*** *and AFM images* ***(b)*** *of a few-layer BP flake prior to the laser irradiation confirm its pristine nature. A corresponding height profile* ***(c)*** *extracted across the white line marked in* ***(b)*** *reveals its initial thickness. The high peak in the height profile corresponds to a thick BP nanosheet lying on the observed flake, as likewise seen due to the light contrast in the AFM image at that position, which was cut in the spectrum for better visibility of the original flake thickness. Optical* ***(d)*** *and AFM images* ***(e)*** *of the identical BP flake after locally controlled laser irradiation. Horizontal rectangular regions of the BP nanosheet were exposed to a 532 nm laser with varying laser powers up to 15 mW (see* ***Figure S1*** *for detailed experimental parameters). For comparison, a second height profile was extracted after the laser exposure* ***(f)*** *from the same position indicated in* ***(e)****, illustrating the changes in topography after the laser exposure with 15 mW.*

S4: Reference experiment on around 10 nm thin BP flakes towards their tendency for photo-induced oxidation

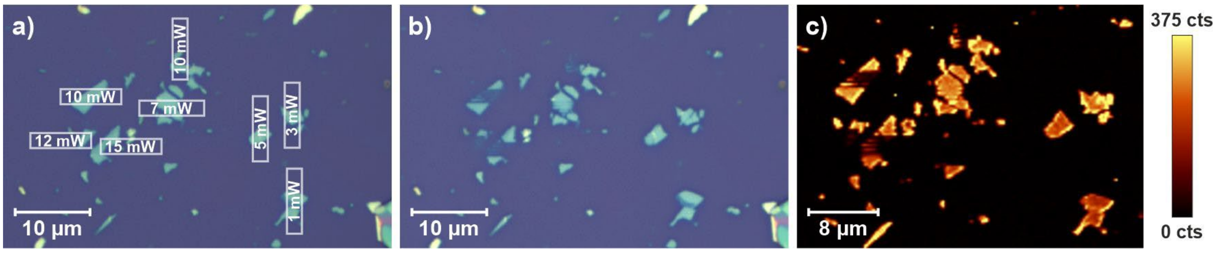

Figure S4. *Laser irradiation of various thin BP flakes using varying laser powers between 1 mW and 15 mW, revealing optical changes before* ***(a)*** *and after irradiation* ***(b)****. The same can be observed in the Raman mapping depicting the* A^1_g *band of BP* ***(c)****, confirming the decrease or disappearance of the characteristic BP Raman bands after high laser power irradiation.*

Overall, eight BP flakes with roughly the same height and in close proximity to each other were selected on the same sample. The areas, that were irradiated with a green laser, are marked with a white rectangle. As the selected flakes were rather small in dimensions, each flake was irradiated with one specific set of parameters, identical to those used in **Figure S1**. All writing parameters were kept constant (532 nm laser wavelength, 3 s irradiation time, 0.5 µm step size between irradiated points) except for the exact length of the lines due to the distinct shape of the irradiated BP flakes and the corresponding laser power, indicated in the respective white rectangular. The optical image in (a) shows the flakes irradiated in the laser power regime between 1 mW and 15 mW. Similarl to **Figure 1** in the manuscript and **Figure S3**, starting from a certain laser power threshold, dark lines appear after the irradiation.

S5: Irradiation of a BP nanosheet using a 633 nm laser

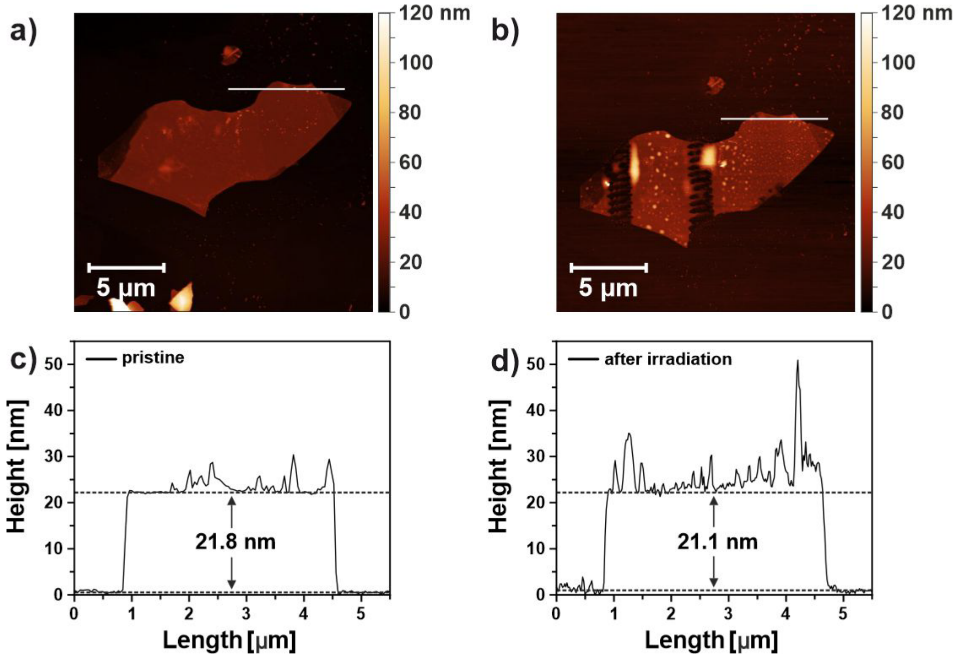

***Figure S5**. AFM height comparison of a BP flake irradiated with three laser wavelengths. AFM images before **(a)** and after irradiation **(b)** show the impact of laser irradiation with different wavelengths of 457 nm (left 'burnt' line), 532 nm (middle line) and 633 nm (right line). BP flake alterations after irradiation with the red laser are minimal, however, height profiles before **(c)** and after the laser irradiation **(d)** extracted along the marked lines in **(a)** and **(b)** reveal a diminished height in the border regions of the flake.*

S6: Energy-dispersive X-ray mapping of a BP flake after the laser-triggered covalent functionalization using Cl-DBPO

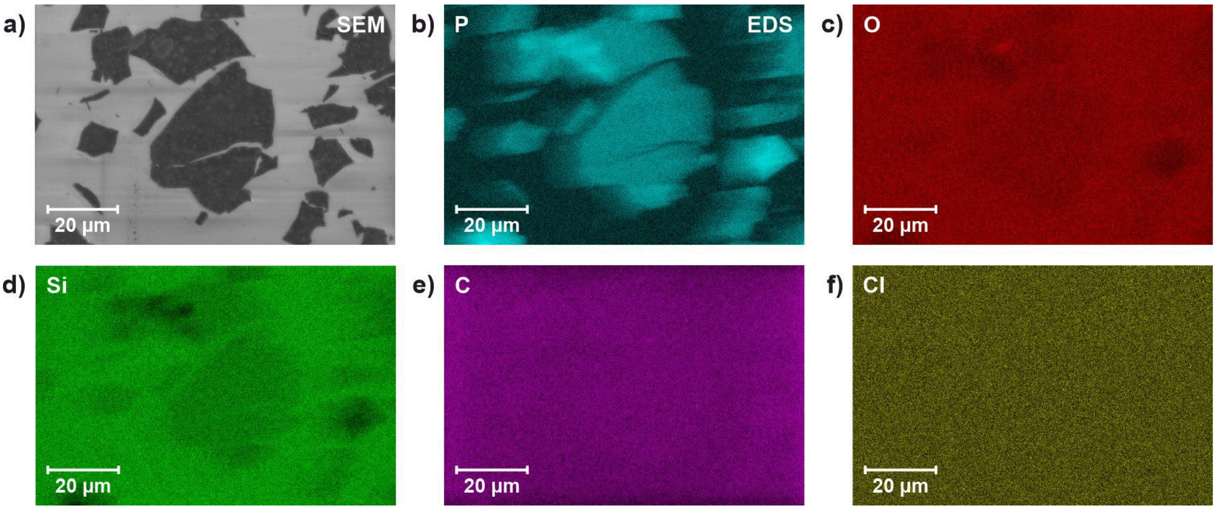

Figure S6. *Recorded scanning electron (SEM) image* ***(a)*** *of an irradiated BP flake after the direct laser writing approach. Corresponding energy-dispersive X-ray (EDX) mappings depicting the distribution of pre-selected elements, in this case of phosphorus* ***(b)****, oxygen* ***(c)****, silicon* ***(d)****, carbon* ***(e)*** *and chlorine* ***(f)****.*

S7: Mean Raman spectra of the pristine G-BP heterostructure under mild laser irradiation conditions

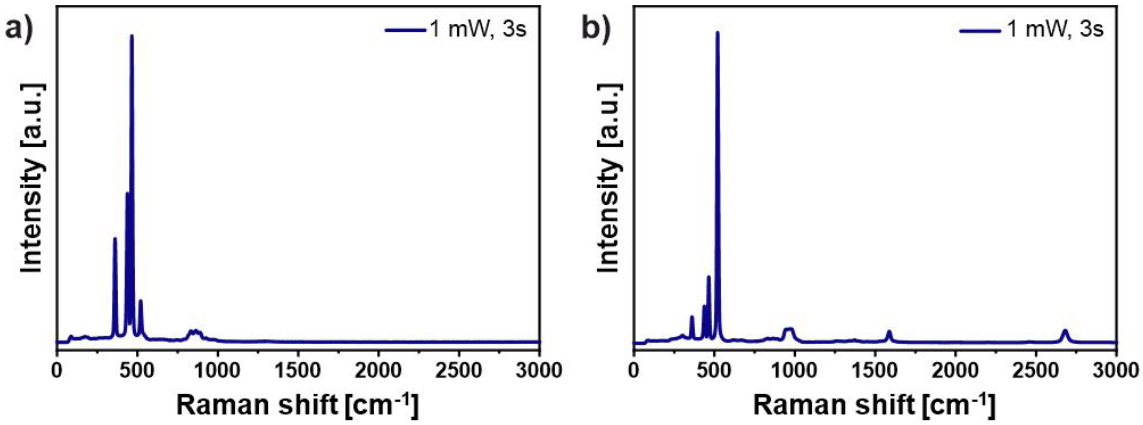

Figure S7. *Mean Raman spectra extracted from the G-BP heterostructure presented in Figure 8 of the manuscript, showing the G-BP heterostructure region (a) and the entire irradiated region (b) when using a laser power of 1 mW and 3 s integration time.*

The presented extracted mean Raman spectra from the experiment performed and presented in Figure 8 of the manuscript confirm, that the observed emergence of a pronounced *D* band can solely be observed in the presence of the reactive precursor Cl-DBPO.

S8: Single Raman spectrum of the pristine G-BP heterostructure

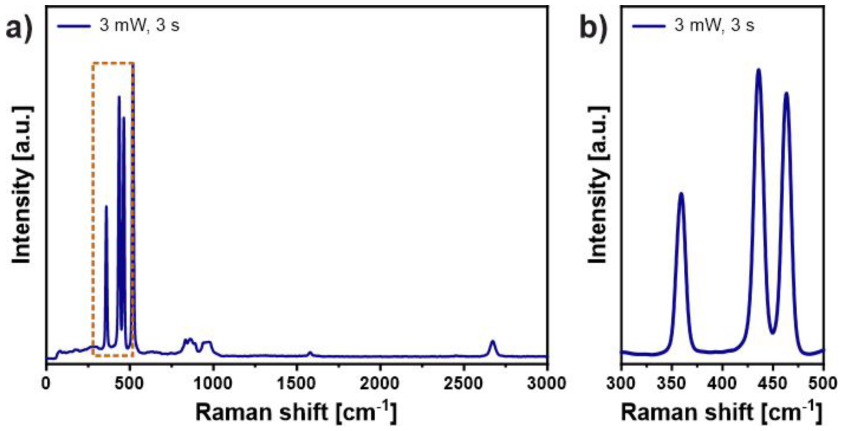

Figure S8. *Single-point Raman spectrum of the G-BP heterostructure **(a)** shown in **Figure 9** and **Figure 10** in the manuscript before the covalent functionalization. The orange-marked spectral region in **(a)** is further zoomed-in in **(b)** for better visibility of the characteristic BP Raman bands.*

Theoretical calculations

Calculations at the four-interface system

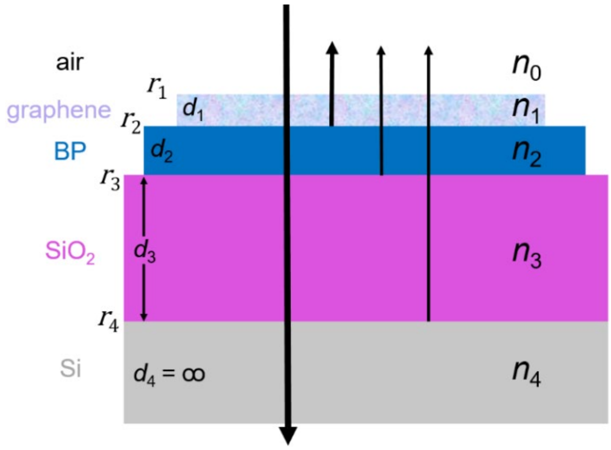

Scheme 1. *Schematic representation taken from [2] of the four-interface system present when measuring Raman spectroscopy of the G-BP heterostructures. The thicknesses* ***d****i, the refractive indices* ***n****i and by which fraction* ***r****i the incident light is reflected, are indicated in the scheme for better visualization. The downward- pointing arrow represents the initial laser irradiation and its intensity (indicated by its thickness), whereas the outgoing arrows correspond to reflections at the respective interfaces.*

The intensity of the reflected laser light I(R) can be calculated according to Equation (1) (adapted from [3]), when considering a four-interface system

$$I(R) = \left| \frac{r_1 + r_2 e^{-i\Delta_1} + r_3 e^{-i(\Delta_1+\Delta_2)} + r_4 e^{-i(\Delta_1+\Delta_2+\Delta_3)} + r_1 r_2 r_3 e^{-i\Delta_2} + r_1 r_3 r_4 e^{-i\Delta_3} + r_1 r_2 r_4 e^{-i(\Delta_2+\Delta_3)} + r_2 r_3 r_4 e^{-i(\Delta_1+\Delta_2)}}{1 + r_1 r_2 e^{-i\Delta_1} + r_1 r_3 e^{-i(\Delta_1+\Delta_2)} + r_1 r_4 e^{-i(\Delta_1+\Delta_2+\Delta_3)} + r_2 r_3 e^{-i\Delta_2} + r_3 r_4 e^{-i\Delta_3} + r_2 r_4 e^{-i(\Delta_2+\Delta_3)} + r_1 r_2 r_3 r_4 e^{-i(\Delta_1+\Delta_3)}} \right|^2 \quad (1)$$

whereas the fractions of the reflected light r_i are defined as

$$r_1 = \frac{n_{air} - n_{graphene}}{n_{air} + n_{graphene}} \qquad r_2 = \frac{n_{graphene} - n_{BP}}{n_{graphene} + n_{BP}}$$

$$r_3 = \frac{n_{BP} - n_{SiO_2}}{n_{BP} + n_{SiO_2}} \qquad r_4 = \frac{n_{SiO_2} - n_{Si}}{n_{SiO_2} + n_{Si}}$$

and the path differences Δ_i of the reflected partial waves are defined as

$$\Delta_1 = \frac{4\pi}{\lambda} n_{graphene} d_{graphene} \qquad \Delta_2 = \frac{4\pi}{\lambda} n_{BP} d_{BP} \qquad \Delta_3 = \frac{4\pi}{\lambda} n_{SiO_2} d_{SiO_2}$$

The contrast C, defined as the relative intensity of the reflectance with and without graphene, can then subsequently be calculated according to Equation (2)[4]

$$C = \frac{I(R, n_{graphene} = 1) - I(R, n_{graphene})}{I(R, n_{graphene} = 1)} \quad (2)$$

In the abovementioned equations, the following wavelength-dependent refractive indices for the indicated material at λ = 532 nm were used:

$n_{Si} = 4.21 - 0.01i$ [5] $\qquad n_{SiO_2} = 1.46$ [5] $\qquad n_{BP} = 3.62 - 0.05i$ [6] $\qquad n_{graphene} = 2.6 - 1.3i$ [4]

References

[1] T. Nagel, S. Wolff, S. Feng, H. Weber, J. Maultzsch, F. Hauke, A. Hirsch, "Towards Precision Controlled 2D Functional Group Patterning of Graphene via Laser Writing" *Carbon* **2025**, *241*, 120376.

[2] T. Dierke, PhD thesis, Friedrich-Alexander-University Erlangen-Nürnberg, **2025**.

[3] H. Anders, *Duenne Schichten Fuer Die Optik*, Wissenschaftliche Verlagsgesellschaft Stuttgart, **1965**.

[4] P. Blake, E. W. Hill, A. H. Castro Neto, K. S. Novoselov, D. Jiang, R. Yang, T. J. Booth, A. K. Geim, "Making Graphene Visible" *Appl. Phys. Lett.* **2007**, *91* (6).

[5] J. Henrie, S. Kellis, S. M. Schultz, A. Hawkins, "Electronic Color Charts for Dielectric Films on Silicon" *Opt. Express* **2004**, *12* (7), 1464.

[6] J. Kim, J.-U. Lee, J. Lee, H. J. Park, Z. Lee, C. Lee, H. Cheong, "Anomalous Polarization Dependence of Raman Scattering and Crystallographic Orientation of Black Phosphorus" *Nanoscale* **2015**, *7* (44), 18708–18715.
